# Transition Metal Complexes Coordinated by Water Soluble Phosphane Ligands: How Cyclodextrins Can Alter the Coordination Sphere?

**DOI:** 10.3390/molecules22010140

**Published:** 2017-01-17

**Authors:** Michel Ferreira, Hervé Bricout, Sébastien Tilloy, Eric Monflier

**Affiliations:** University of Artois, CNRS, Centrale Lille, ENSCL, University of Lille, UMR 8181, Unité de Catalyse et de Chimie du Solide (UCCS), F-62300 Lens, France; michel.ferreira@univ-artois.fr (M.F.); herve.bricout@univ-artois.fr (H.B.); sebastien.tilloy@univ-artois.fr (S.T.)

**Keywords:** platinum, palladium, TPPTS, cyclodextrin, hydrosoluble organometallic complexes, supramolecular chemistry

## Abstract

The behaviour of platinum(II) and palladium(0) complexes coordinated by various hydrosoluble monodentate phosphane ligands has been investigated by ^31^P{^1^H} NMR spectroscopy in the presence of randomly methylated β-cyclodextrin (RAME-β-CD). This molecular receptor can have no impact on the organometallic complexes, induce the formation of phosphane low-coordinated complexes or form coordination second sphere species. These three behaviours are under thermodynamic control and are governed not only by the affinity of RAME-β-CD for the phosphane but also by the phosphane stereoelectronic properties. When observed, the low-coordinated complexes may be formed either via a preliminary decoordination of the phosphane followed by a complexation of the free ligand by the CD or via the generation of organometallic species complexed by CD which then lead to expulsion of ligands to decrease their internal steric hindrance.

## 1. Introduction

Biphasic aqueous organometallic catalysis is one of the greenest solutions to produce organic chemicals [1,2]. Indeed, water is not only cheap and non-toxic, but also permits one to immobilize the catalyst in an aqueous phase by the use of water-soluble ligands, leading to ease of recycling by simple decantation at the end of the reaction [3]. The most widespread ligand used in such systems is TPPTS (tris(3-sulfonatophenyl)phosphane sodium salt, Table 1, entry 1) which is responsible for the industrial success of the aqueous biphasic propene hydroformylation (Ruhrchemie-Rhône Poulenc process, 1984) [4]. Attractive for partially water-soluble olefins, this strategy suffers from low catalytic activity when hydrophobic substrates are used due to mass transfer limitations. The alternative approaches to overcome this drawback have been recently reviewed [5,6]. These include the use of cyclodextrins (CDs) which are widely recognized as outstanding water-soluble receptors in aqueous biphasic organometallic processes [7].

**Scheme 1:**
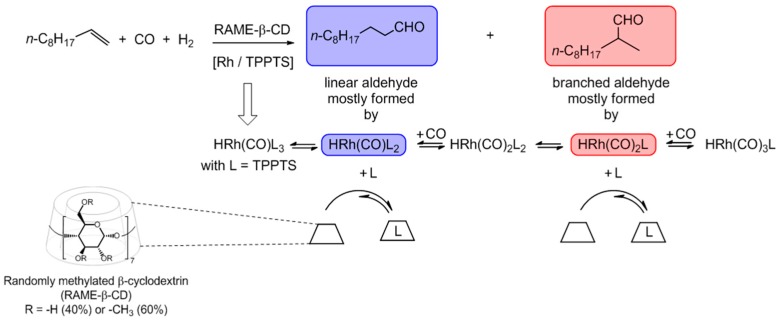
Equilibria between the different catalytic species during 1-decene aqueous biphasic hydroformylation assisted by RAME-β-CD.

These macrocycles were firstly used as phase transfer agents between the organic and the aqueous layers by forming inclusion complexes with a hydrophobic substrate at the interface in order to convert it thanks to a water-soluble organometallic catalyst. However, CDs are more than simple receptors for hydrophobic substrates and can be now considered as polyfunctional entities in aqueous biphasic organometallic catalysis [8]. In particular, CDs can modify the catalytic species involved in the reaction. As an example, when TPPTS is combined with a rhodium precursor to catalyse the 1-decene aqueous biphasic hydroformylation reaction, a linear to branched aldehyde ratio drop is observed when the experiment is conducted in the presence of RAME-β-CD (=randomly methylated β-CD) [9]. Under CO/H_2_ pressure and with RAME-β-CD, equilibria displacements between the different catalytic species towards the formation of phosphane low-coordinated species are observed. These equilibria, displaced by TPPTS inclusion into the CD cavity, are responsible for the drop in regioselectivity (Scheme 1) [10].

Along the same lines, when a water-soluble triphenylphosphane derivative possessing a higher affinity towards CDs is involved in a rhodium HRhCOL_3_ type species (L = *p*-tBuPhP(*m*-PhSO_3_Na)_2_ phosphane), unsaturated catalytic species are also formed in the presence of CD even when the organometallic complex was not exposed to a CO/H_2_ atmosphere [11].

This behaviour, not observed with TPPTS, was attributed to the more stable inclusion complex formed between the CD and the phosphane, due to the presence of the well-recognized *p*-*tert*-butylphenyl group. Moreover, it has been demonstrated that the combination of the same ligand with another metal centre, e.g., palladium or platinum, led to the same behaviour, that is to say formation of low-coordinated organometallic species [12].

However, the combination of a palladium precursor with a water-soluble triphenylphosphane derivative possessing a *p*-*tert*-butylbiphenyl group led to the formation of a stable second-sphere coordination adduct where the CD encapsulated the ligand directly bounded to the metal centre [13]. Such species had already been observed with a phosphane bearing adamantyl groups [14].

In order to get a better insight into the interaction phenomena between the CD and transition metal complexes, we want to show in this article how RAME-β-CD can interfere with different platinum(II) or palladium(0) complexes coordinated with various hydrosoluble ligands and how specific organometallic complexes can be obtained. These ligands include TPPTS itself but also TPPTS modified phosphanes containing substituents (methyl or methoxy groups) in *ortho* or *para* position and biphenyl modified phosphanes (Table 1). The interaction of the different phosphanes with RAME-β-CD has already been studied by our group [15], except for biphenylphosphane derivatives whose synthesis and catalytic properties have been exclusively published [16]. For each type of ligands, we have both examined the nature of the organometallic complex formed when mixing them with the metal precursor and the influence of the RAME-β-CD addition on these complexes.

## 2. Results and Discussion

### 2.1. Platinum Complexes

The different complexes formed by mixing the K_2_PtCl_4_ aqueous solution with 3 equiv. of phosphane were firstly analysed by ^31^P{^1^H} NMR. In a second step, RAME-β-CD has been added to the mixture in order to determine its influence on the coordination chemistry of the different ligands.

#### 2.1.1. Complex Synthesis

##### TPPTS Ligand

**Figure 1:**
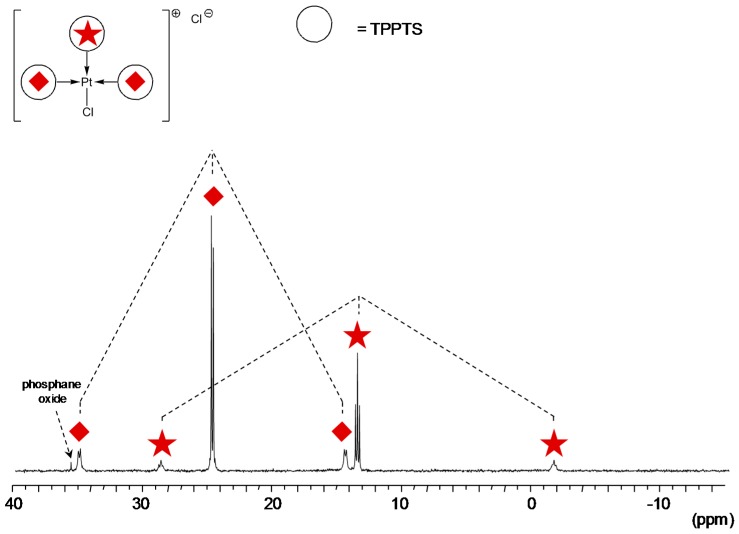
^31^P{^1^H} NMR spectrum of [Pt(TPPTS)_3_Cl]Cl, [Pt] = 10 mM, 25 °C in D_2_O.

As described in the literature [17], [Pt(TPPTS)_3_Cl]Cl platinum complex is quantitatively synthetized by adding three equivalents of TPPTS to a K_2_PtCl_4_ aqueous solution. As only one third of the platinum (^195^Pt) has a spin of 1/2, the ^31^P{^1^H} NMR spectrum of this complex is characterized by a 1:4:1 triplet of doublets centred at δ = 24.5 ppm (^1^*J*P*_cis_*–Pt = 2491 Hz; ^2^*J*P*_cis_*–P*_trans_* = 19 Hz) corresponding to the two phosphorus atoms *cis* to the chloride and a 1:4:1 triplet of triplets centred at δ = 13.3 ppm (^1^*J*P*_trans_*–Pt = 3682 Hz; ^2^*J*P*_cis_*–P*_trans_* = 19 Hz) corresponding to the phosphorus atom *trans* to the chloride (Figure 1).

##### *para*-Substituted TPPTS Derivatives

**Figure 2:**
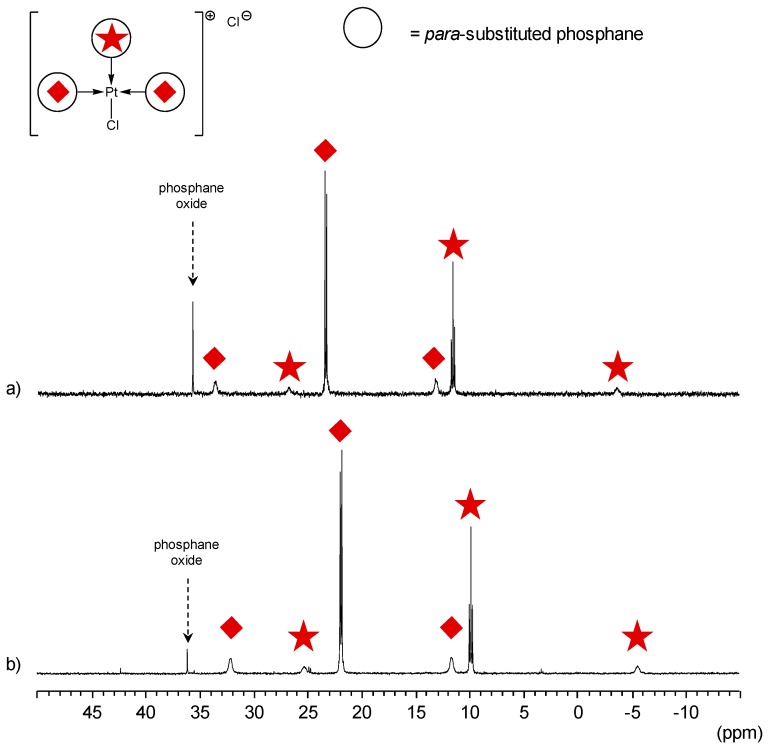
^31^P{^1^H} NMR spectra of (**a**) [Pt(tris(*p*-Me)TPPTS)_3_Cl]Cl and (**b**) [Pt(tris(*p*-OMe)TPPTS)_3_Cl]Cl, [Pt] = 10 mM, 25 °C in D_2_O.

Similarly to TPPTS, [PtL_3_Cl]Cl platinum complexes can be quantitatively synthetized by adding three equivalents of the *para*-substituted ligands tris(*p*-Me)TPPTS and tris(*p*-OMe)TPPTS to a solution of K_2_PtCl_4_ (Figure 2). The two phosphorus atoms *cis* to the chloride appear respectively for [Pt(tris(*p*-Me)TPPTS)_3_Cl]Cl and [Pt(tris(*p*-OMe)TPPTS)_3_Cl]Cl at δ = 23.3 ppm (^1^*J*P*_cis_*–Pt = 2488 Hz; ^2^*J*P*_cis_*–P*_trans_* = 19 Hz) and δ = 21.8 ppm (^1^*J*P*_cis_*–Pt = 2487 Hz; ^2^*J*P*_cis_*–P*_trans_* = 18 Hz) in their ^31^P{^1^H} NMR spectra. The phosphorus atom *trans* to the chloride appears at δ = 11.5 ppm (^1^*J*P*_trans_*–Pt = 3693 Hz; ^2^*J*P*_cis_*–P*_trans_* = 19 Hz) and δ = 9.8 ppm (^1^*J*P*_trans_*–Pt = 3754 Hz; ^2^*J*P*_cis_*–P*_trans_* = 18 Hz).

##### *ortho*-Substituted TPPTS Derivatives

**Figure 3:**
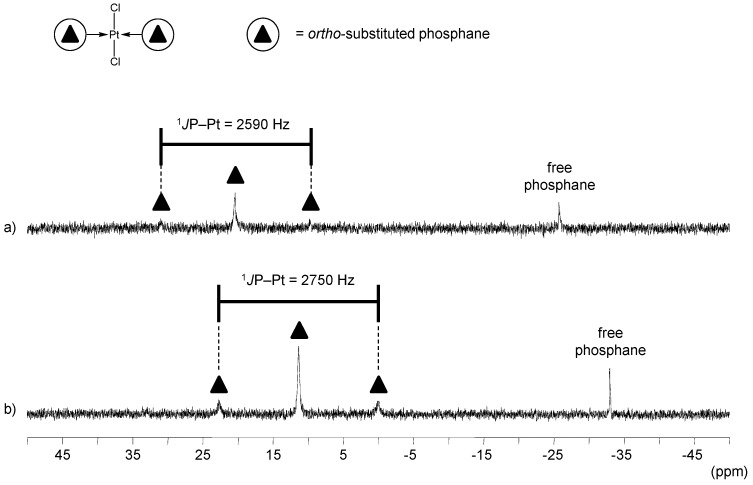
^31^P{^1^H} NMR spectra of K_2_PtCl_4_ (3 mM) + 3 equiv. of (**a**) tris(*o*-Me)TPPTS or (**b**) tris(*o*-OMe)TPPTS at 25 °C in D_2_O.

**Figure 4:**
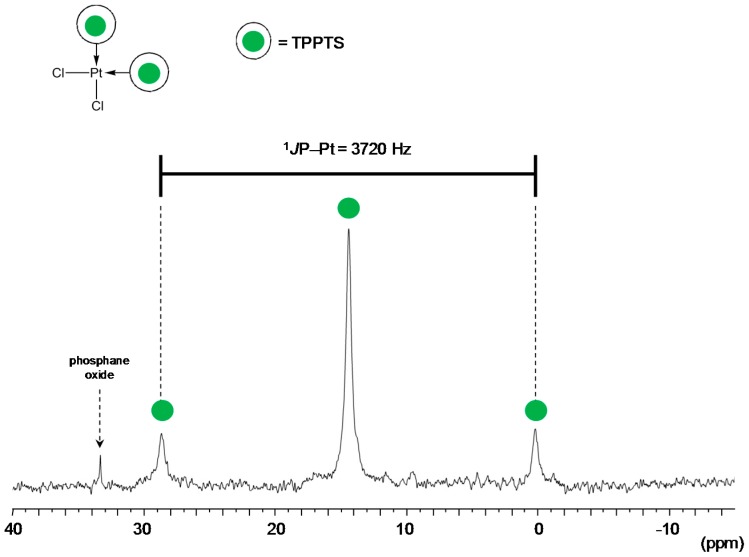
^31^P{^1^H} NMR spectrum of *cis*-PtCl_2_(TPPTS)_2_, [Pt] = 10 mM, 25 °C in D_2_O.

However, attempts to synthetize [Pt(tris(*o*-Me)TPPTS)_3_Cl]Cl and [Pt(tris(*o*-OMe)TPPTS)_3_Cl]Cl were unsuccessful. Indeed, addition of three equivalents of ligand (towards platinum) to a K_2_PtCl_4 _solution exclusively yields to formation of PtCl_2_L_2_-type complex characterized by a 1:4:1 triplet of doublets centred at δ = 20.5 ppm and 24.5 ppm, respectively, for PtCl_2_(tris(*o*-Me)TPPTS)_2_ and PtCl_2_(tris(*o*-OMe)TPPTS)_2_. As a consequence, free ligand that represents one third of the total ^31^P{^1^H} NMR surface area is detected (Figure 3). Moreover, in both cases, ^1^*J*P–Pt coupling constants (2750 and 2590 Hz for PtCl_2_(tris(*o*-OMe)TPPTS)_2_ and PtCl_2_(tris(*o*-Me)TPPTS)_2_ respectively) are consistent with a *trans* coordination when compared with *cis*-PtCl_2_(TPPTS)_2_ (^1^*J*P–Pt = 3720 Hz, Figure 4) and *trans*-PtCl_2_(TPPTS)_2_ (^1^*J*P–Pt = 2600 Hz) that have already been described [17]. The steric hindrance of these ligands, which have a higher cone angle than their *para*-substituted counterparts, is obviously responsible for the formation of the observed phosphane low coordinated PtCl_2_L_2_ species. The same reason can also explain the *trans* geometry obtained for these species instead of the *cis* one [18].

##### Trisulfonated Biphenylphosphane Ligands

**Figure 5:**
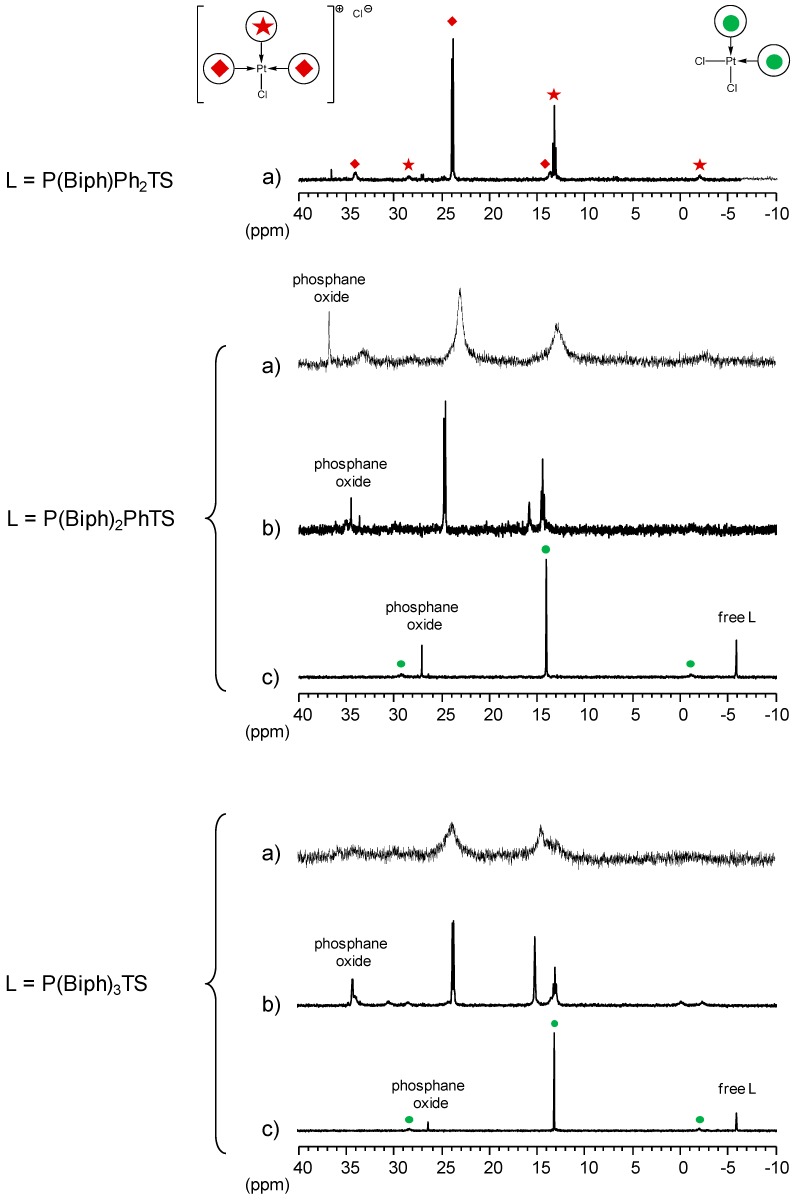
^31^P{^1^H} NMR spectra of K_2_PtCl_4_ solution (10 mM) + 3 equiv. of ligand (L) at 25 °C (**a**) in D_2_O (**b**) in D_2_O/DMSO-*d_6_* mixture (50/50 vol.) (**c**) in DMSO-*d_6_*.

Again, [PtL_3_Cl]Cl complex was obtained by adding three equivalents of the monobiphenyl ligand P(Biph)Ph_2_TS to a K_2_PtCl_4_ aqueous solution. However, when P(Biph)_2_PhTS and P(Biph)_3_TS were used, the complexes appeared as broad signals in the ^31^P{^1^H} NMR spectra. Addition of DMSO to the previous aqueous solutions achieves finer signals (Figure 5).

In pure DMSO, *cis*-PtCl_2_L_2_ is formed quantitatively (^1^*J*P–Pt = 3696 Hz when L = P(Biph)_2_PhTS and ^1^*J*P–Pt = 3702 Hz when L = P(Biph)_3_TS). The lowest DMSO polarity compared to water is probably responsible for the formation of these non-ionic complexes. When the water quantity of the medium is increased, the polarity increases as well probably leading to the formation of [PtL_3_Cl]Cl. With these two ligands, fast exchange between [PtL_3_Cl]Cl complex and water molecules would be responsible for the signals broadening.

#### 2.1.2. RAME-β-CD Effects on the Coordination Behaviour of the Ligands

As a reminder of our previous work, Table 1 brings together the association constants between the different hydrosoluble ligands studied and the CDs [15].

##### TPPTS Ligand

**Figure 6:**
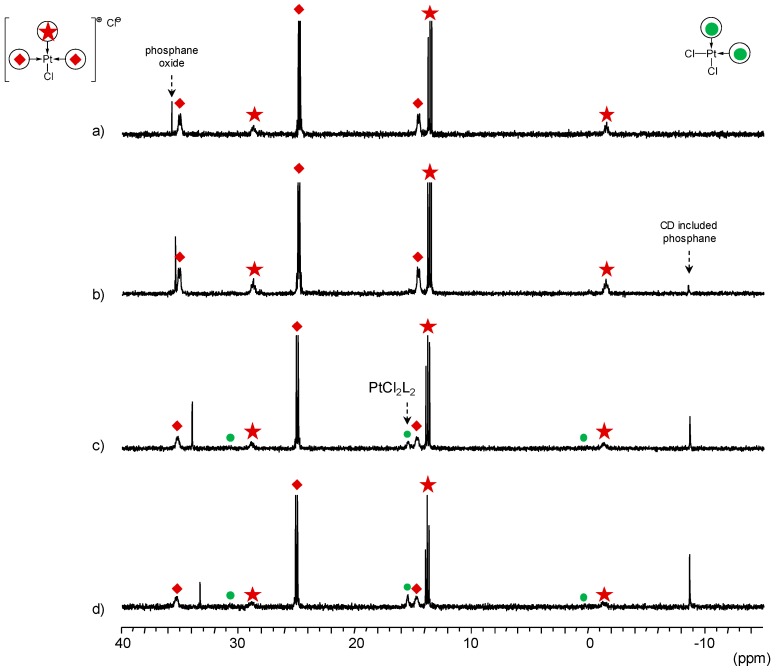
^31^P{^1^H} NMR spectra of (**a**) [Pt(TPPTS)_3_Cl]Cl (10 mM); (**b**) +3 equiv. of RAME-β-CD toward Pt (**c**) +6 equiv. of RAME-β-CD and (**d**) +12 equiv. of RAME-β-CD at 25 °C in D_2_O.

When 1equiv. of RAME-β-CD toward platinum was added to a solution of the [Pt(TPPTS)_3_Cl]Cl complex, a downfield chemical shift of the TPPTS oxide signal was observed and attributed to the formation of an inclusion complex with RAME-β-CD [19]. Moreover, a typical signal of CD-included TPPTS appeared at −8.8 ppm as well as a *cis*-PtCl_2_(TPPTS)_2_ signal at 15.5 ppm (Figure 6).

**Scheme 2:**
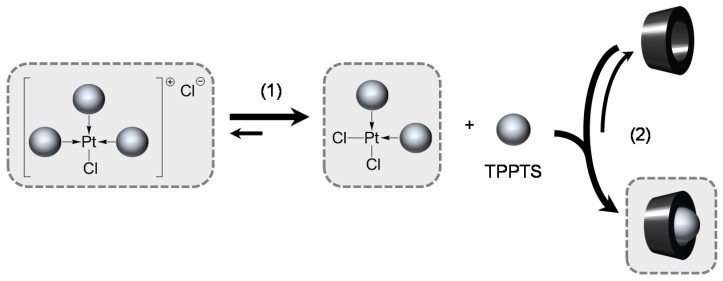
Equilibria between the different platinum species with TPPTS as ligand in the presence of RAME-β-CD (species observed on the ^31^P{^1^H} NMR spectrum are presented on a grey background).

By adding increasing amounts of CD (3 to 12 equiv.), these signals are intensified. Thus, RAME-β-CD is able to modify the equilibrium between these two organometallic species. In particular, the formation of phosphane low-coordinated organometallic species is favoured by the formation of an inclusion complex between RAME-β-CD and TPPTS as depicted in Scheme 2 (equilibrium (1) is displaced by equilibrium (2)). Note that such behaviour is similar to what is observed by high pressure NMR when RAME-β-CD is added, under hydroformylation conditions (50 bars CO/H_2_ and 80 °C), on the HRh(CO)(TPPTS)_3_ rhodium complex. In that case, HRh(CO)_2_(TPPTS)_2_ species combined with TPPTS included in the CD cavity were highlighted [10].

**Figure 7:**
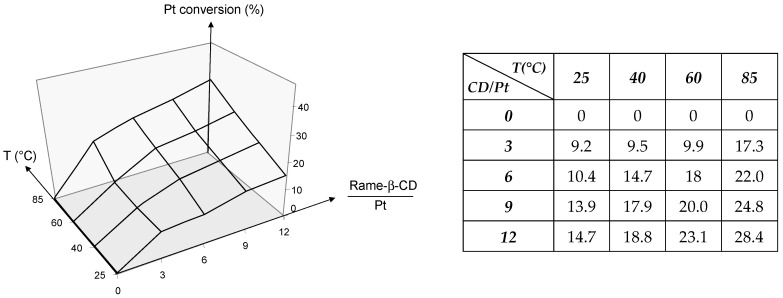
[Pt(TPPTS)_3_Cl]Cl conversion (%) into *cis*-PtCl_2_(TPPTS)_2_ as a function of RAME-β-CD quantity and temperature, [Pt] = 10 mM, D_2_O.

The conversion of [Pt(TPPTS)_3_Cl]Cl into *cis*-PtCl_2_(TPPTS)_2_ as a function of the RAME-β-CD quantity (Figure 7) can be measured by integration of the ^31^P{^1^H} NMR spectra (see ESI). As expected, the conversion increases with the quantity of RAME-β-CD in solution. The temperature also has an influence on these equilibria. Indeed, the conversion increases with the temperature, therefore, implying that the natural dissociation of TPPTS from [Pt(TPPTS)_3_Cl]Cl is more affected compared to the inclusion complex stability. Moreover, the NMR spectrum carried out at room temperature after heating gave back the signals with the same initial intensity reflecting the process reversibility and assuming a thermodynamic control of the system.

##### *para*-Substituted TPPTS Derivatives

The behaviour of tris(*p*-OMe)TPPTS and tris(*p*-Me)TPPTS towards RAME-β-CD is different. Indeed, unlike tris(*p*-OMe)TPPTS, tris(*p*-Me)TPPTS forms an inclusion complex with this CD (K = 960 M^−1^) [15]. The highest association constant of this inclusion complex compared to the complex RAME-β-CD/TPPTS (K = 840 M^−1^) is due to the deep inclusion of the tris(*p*-Me)TPPTS via one of its aryl groups into the CD cavity [20].

**Scheme 3:**
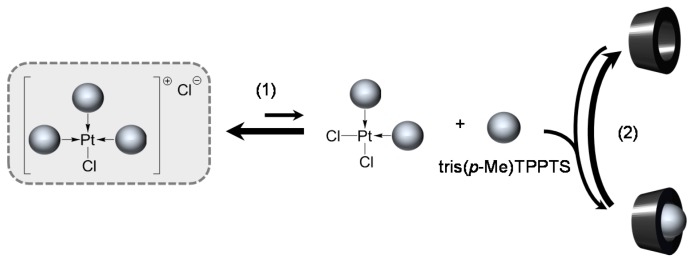
Equilibria between the different platinum species with tris(*p*-Me)TPPTS as ligand in the presence of RAME-β-CD (species observed on the ^31^P{^1^H} NMR spectrum are presented on a grey background).

Given that the tris(*p*-OMe)TPPTS does not interact with RAME-β-CD, it is not surprising to see no modifications on the ^31^P{^1^H} NMR spectrum of the [Pt(tris(*p*-OMe)TPPTS)_3_Cl]Cl organometallic complex when the RAME-β-CD was added (see ESI). Surprisingly, the same behaviour was observed with the tris(*p*-Me)TPPTS ligand. Indeed, although the association constant between RAME-β-CD and tris(*p*-Me)TPPTS is higher than the one between RAME-β-CD and TPPTS, no conversion of [Pt(tris(*p*-Me)TPPTS)_3_Cl]Cl to PtCl_2_(tris(*p*-Me)TPPTS)_2_ was highlighted after the addition of RAME-β-CD, even at high temperature (see ESI) [21]. This result can be explained by the higher σ-donating strength of tris(*p*-Me)TPPTS ligand, compared to TPPTS. Consequently, the equilibrium (1) is shifted towards the formation of the high coordinated platinum species [Pt(tris(*p*-Me)TPPTS)_3_Cl]Cl even in the presence of RAME-β-CD (Scheme 3).

##### *Ortho*-Substituted TPPTS Derivatives

Since the *ortho*-substituted ligands do not interact with RAME-β-CD [15,20], addition of CD did not modify the ^31^P{^1^H} NMR spectra.

##### Trisulfonated Biphenylphosphane Ligands

Phosphanes possessing one, two or three sulfonated biphenyl groups strongly interact with the β-CD. Indeed, complexation studies between these ligands and native β-CD showed a deep inclusion of their biphenyl moiety in the CD cavity. In all cases, stoichiometries were determined to be equal to 1:1 and association constants were particularly high (Table 1 and Supplementary Materials). Consequently, the influence of such association towards the platinum organometallic complexes was investigated.

**Figure 8:**
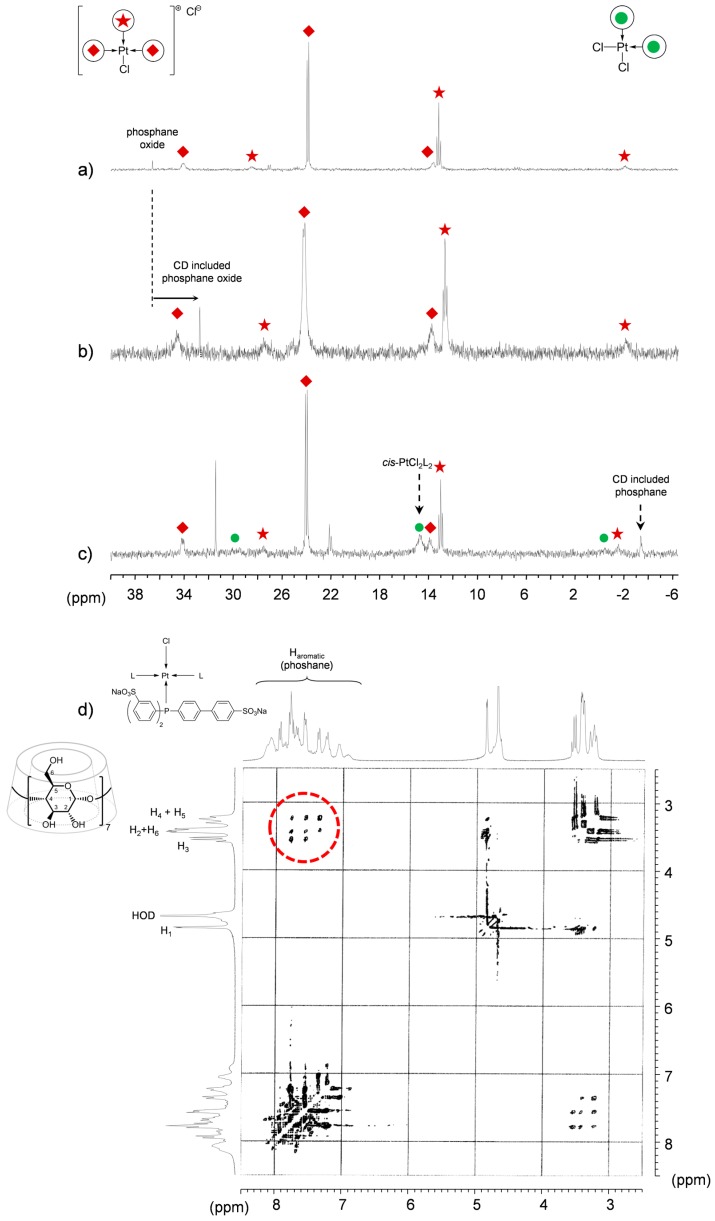
^31^P{^1^H} NMR spectra of (**a**) [Pt(P(Biph)Ph_2_TS)_3_Cl]Cl 10 mM; (**b**) +3 equiv. of RAME-β-CD toward Pt at 25 °C (**c**) +12 equiv. of RAME-β-CD toward Pt at 85 °C (**d**) 2D T-ROESY spectrum of a solution containing β-CD (10 mM) and [Pt(P(Biph)Ph_2_TS)_3_Cl]Cl (10 mM) at 25 °C in D_2_O.

**Scheme 4:**
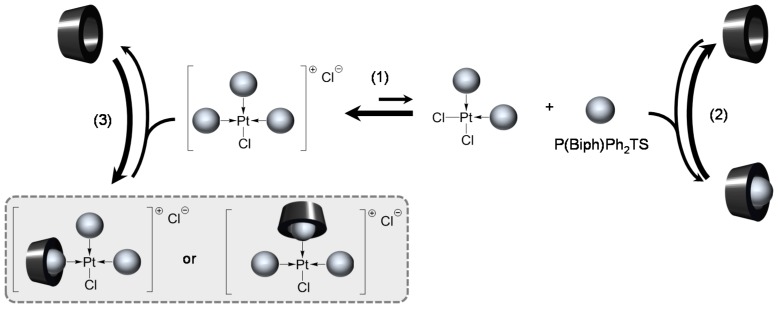
Equilibria between the different platinum species with P(Biph)Ph_2_TS as ligand in the presence of 3 equiv. of RAME-β-CD toward Pt (species observed on the ^31^P{^1^H} NMR spectrum are presented on a grey background).

In the case of P(Biph)Ph_2_TS, modifications on the ^31^P{^1^H} NMR spectra are observed with RAME-β-CD addition. Complexation of the phosphane oxide by the CD is in fact detected by its chemical shift modification. Moreover, a broadening of [PtL_3_Cl]Cl signals is observed concurrently with the lack of peak corresponding to free or CD-complexed phosphane (in the region going from 0 to −10 ppm) (Figure 8). The explanation for that is the interaction between RAME-β-CD and [Pt(P(Biph)Ph_2_TS)_3_Cl]Cl without inducing dissociation of the ligand from the metal centre. This theory was confirmed by the 2D T-ROESY experiment of a 1:1 β-CD:[Pt(P(Biph)Ph_2_TS)_3_Cl]Cl [22] mixture which exhibited intense cross-peaks between the phosphane aromatic protons and the internal CD protons (H_3_ and H_5_). In that case, an organometallic complex formation where the CD plays the role of coordination second sphere ligand is highlighted (Scheme 4). Thus, contrary to TPPTS, three equivalents of RAME-β-CD with respect to Pt are not able to transform the [PtL_3_Cl]Cl complex into PtCl_2_L_2_ complex when P(Biph)Ph_2_TS is used. Nevertheless, further increase in the CD quantity (12 equivalents of RAME-β-CD relative to Pt) and temperature (85 °C) lead to a dissociation of the ligand from the metal centre confirmed both by the CD-included phosphane signal observed at −3 ppm and the formation of low-coordinated PtCl_2_L_2_ species (Figure 8c).

On the other hand, when P(Biph)_2_PhTS and P(Biph)_3_TS were used, addition of large amounts of CD to the complexes synthetized by adding three equivalents of the previous ligands to a K_2_PtCl_4_ aqueous solution did not lead to a signal improvement (obtained spectra were comparable to those presented in Figure 5(a)), so in these cases, it is difficult to conclude.

A short overview of this platinum study is presented in Table 2. To sum up, low-coordinated catalytic species PtCl_2_L_2_ are formed when *ortho*-substituted TPPTS derivatives are used or when TPPTS-based complexes are combined with RAME-β-CD (*trans* and *cis* geometry respectively). In the first case, these species are obviously formed due to the larger cone angles of these *ortho*-substituted ligands. In the second case, the relative σ-donating power of the TPPTS ligand, through the presence of the attractive *meta* sodium sulfonato groups, makes the ligand-metal bond weak and hence RAME-β-CD is able to shift the equilibria between the different organometallic species towards the formation of low-coordinated species by the formation of inclusion complex with the ligand. However, although the association constant between tris(*p*-Me)TPPTS and RAME-β-CD is higher than the one between TPPTS and RAME-β-CD, low-coordinated platinum complexes are not observed. 

This behaviour comes from the highest σ-donating power of this ligand compared to TPPTS through the presence of the donating *para* methyl groups which makes the ligand-metal bond stronger. Furthermore, given that tris(*p*-OMe)TPPTS does not interact with RAME-β-CD, its coordination behaviour towards platinum(II) is not affected by the CD addition. Finally, formation of organometallic species where the RAME-β-CD plays the role of coordination second sphere ligand is observed when P(Biph)Ph_2_TS, which possesses a CD-recognised moiety, is used in combination with one equivalent of RAME-β-CD towards the ligand. It is important to emphasize that further increase in the CD quantity yields to the formation of low-coordinated species. The formation of organometallic species possessing more than one CD in their coordination second sphere would probably yield to a steric decompression trough the decoordination of the CD included phosphane.

### 2.2. Palladium Complexes

The RAME-β-CD influence on organometallic species was extended to palladium (0) complexes. The palladium extraction from a toluene solution of Pd(TPP)_4_ to an aqueous phase by the hydrosoluble ligand yields to isolation of PdL*_x_* complexes (see “materials and methods” part). In the case of TPPTS, this study has already been published by our group [19]. It was concluded that the addition of RAME-β-CD only yields to a chemical exchange rate decrease between free TPPTS and the Pd(TPPTS)_3_ complex caused by the formation of the inclusion complex RAME-β-CD/TPPTS. Furthermore, no new palladium species were observed, even at high temperature (60 °C).

#### 2.2.1. *Para*-Substituted TPPTS Derivatives

##### [Pd(0)/tris(*p*-Me)TPPTS] Complex

**Figure 9:**
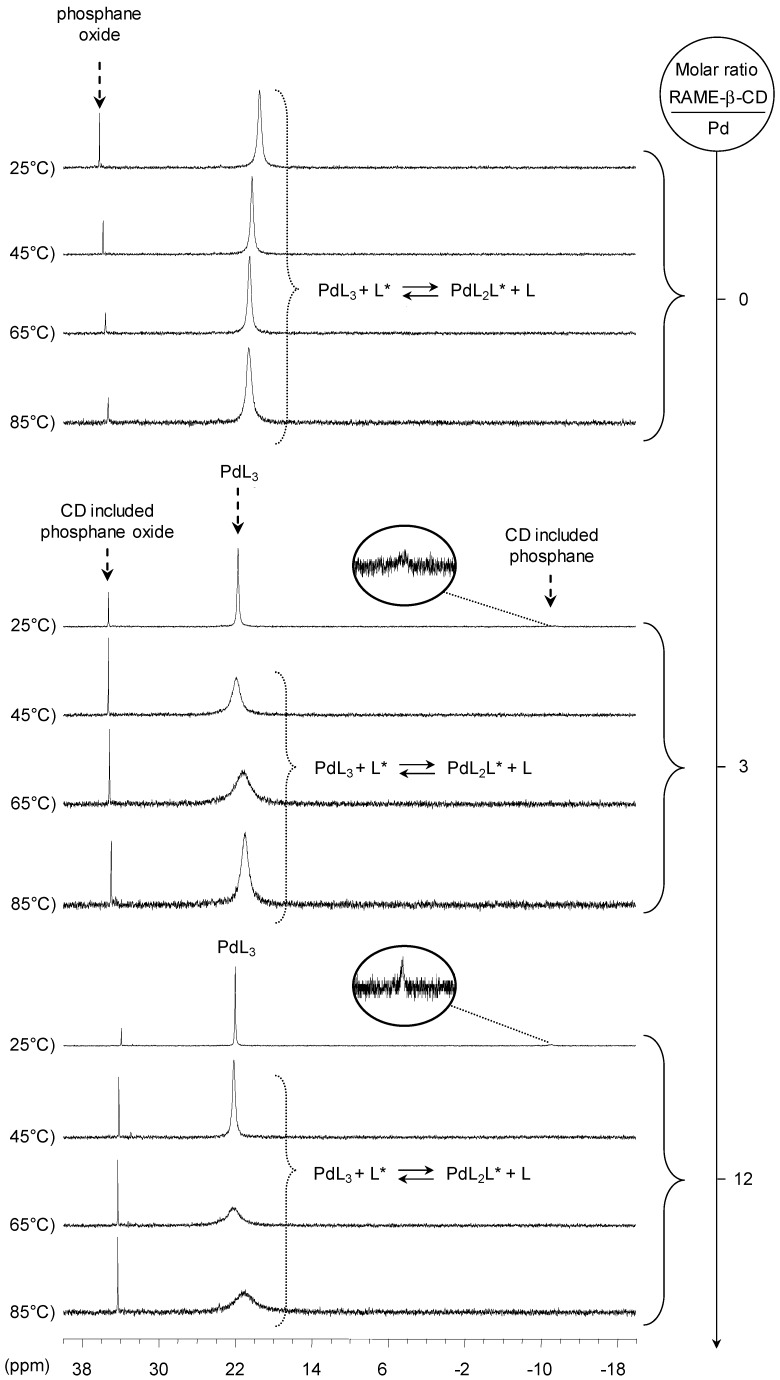
^31^P{^1^H} NMR spectra of Pd(tris(*p*-Me)TPPTS)_3_ depending on both temperature and RAME-β-CD quantity in D_2_O with a ligand concentration of 66 mM.

^31^P{^1^H} NMR spectra of Pd(tris(*p*-Me)TPPTS)_3_ complexes in addition to different RAME-β-CD concentrations and at different temperatures are given in Figure 9. Without CD, two different resonances are observed. Besides the minor phosphane oxide signal at 34 ppm, a broad signal was detected at 19.5 ppm which corresponds to the average signal of the Pd(tris(*p*-Me)TPPTS)_3_ complex in fast equilibrium with a small amount of free residual tris(*p*-Me)TPPTS ligand [19,23]. Higher temperatures have no effects on this equilibrium. Adding three equivalents of RAME-β-CD yields to an exchange rate decrease between the Pd(tris(*p*-Me)TPPTS)_3_ species, which signal becomes sharp at 21.8 ppm, and the small excess of free ligand by its encapsulation in the CD cavity (−11.2 ppm). Higher CD concentrations combined with higher temperatures do not lead to the formation of new palladium species. Hence, like what was observed with the platinum(II) complexes, addition of RAME-β-CD does not change the nature of the organometallic complex, albeit association constant between the CD and the ligand is greater than that of the RAME-β-CD/TPPTS inclusion complex.

##### [Pd(0)/tris(*p*-OMe)TPPTS] Complex

**Figure 10:**
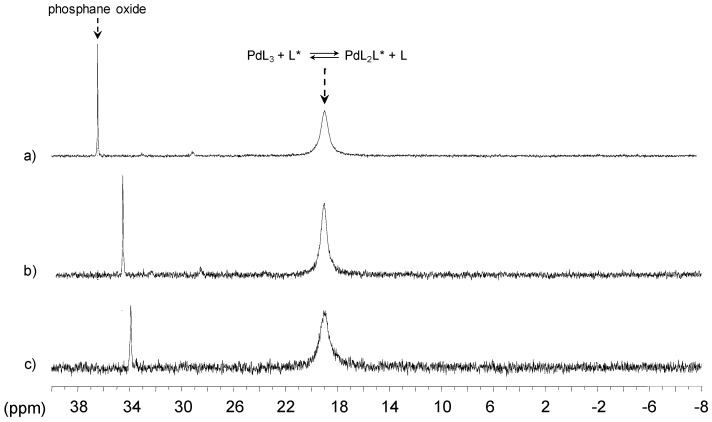
^31^P{^1^H} NMR spectra of Pd(tris(*p*-OMe)TPPTS)_3_ at (**a**) 25 °C; (**b**) +12 equiv. of RAME-β-CD at 25 °C (**c**) +12 equiv. of RAME-β-CD at 85 °C in D_2_O with a ligand concentration of 66 mM.

Addition of large amount of RAME-β-CD (12 equiv. with respect to Pd) to the Pd(tris(*p*-OMe)TPPTS)_3_ complex does not modify the ^31^P{^1^H} NMR spectrum as this ligand does not interact with the CD. In fact, even at high temperature, a broad signal at 19 ppm is detected which corresponds to the average signal of the equilibrium between the organometallic complex and free tris(*p*-OMe)TPPTS (Figure 10).

#### 2.2.2. *ortho*-Substituted Ligands

The same experiments were realized with the *ortho*-substituted ligands but the synthesis of the PdL_3_ complex failed. Indeed, Pd extraction from the organic phase to the aqueous phase is too low. The bulkiness of the hydrosoluble *ortho*-substituted are obviously responsible for the equilibrium displacement toward the organic Pd(TPP)_4_ complex. Again, these results are in accordance with the previous observations made with the platinum complexes study showing that these ligands hardly coordinate the metal centre.

#### 2.2.3. Biphenylphosphane Ligands

##### [Pd(0)/P(Biph)Ph_2_TS] Complex

**Figure 11:**
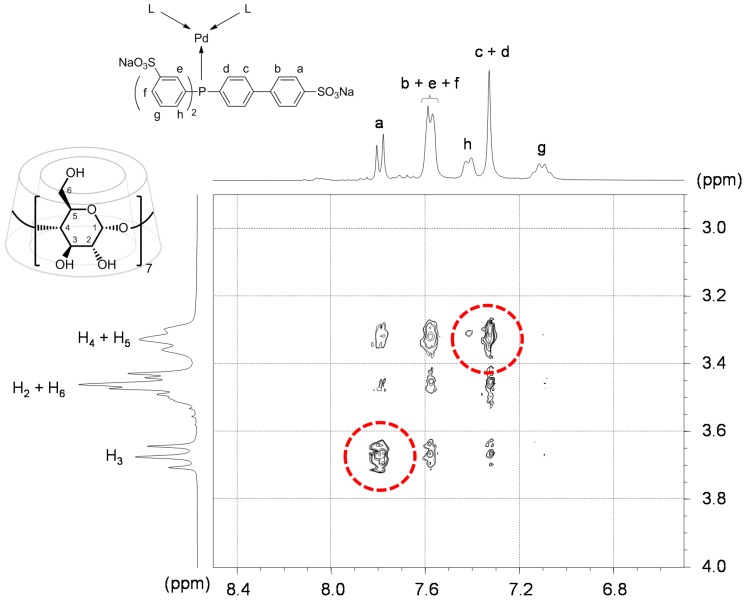
2D T-ROESY spectrum of a solution containing β-CD (11 mM) and Pd(P(Biph)Ph_2_TS)_3_ (11 mM) at 25 °C in D_2_O.

In this case, comments are identical to the previous Pd(tris(*p*-Me)TPPTS)_3_ complex (see ESI). That is to say, CD addition to a Pd(P(Biph)Ph_2_TS)_3_ aqueous solution reduces the chemical exchange rate between free ligand and the organometallic complex. Increasing both temperature and RAME-β-CD quantity do not yield to the formation of other palladium species. However, 2D NMR T-ROESY experiment of a 1:1 β-CD:Pd(P(Biph)Ph_2_TS)_3_ mixture exhibited intense cross-peaks between the phosphane aromatic protons and the internal CD protons H_3_ and H_5_ (Figure 11). The presence of only 4% (determined by integration of the ^31^P{^1^H} NMR spectrum) of free ligand which is trapped by the CD in the mixture could not be responsible for such intense cross peaks. Therefore, absence of new palladium species together with strong correlation between the CD and the ligand in the 2D T-ROESY NMR spectrum enable us to assume the formation of inclusion complex between the CD and the ligand (coordinated to the palladium centre). As with the platinum complex, the P(Biph)Ph_2_TS ligand allows the formation of organometallic palladium species where the cyclodextrin plays the role of coordination second sphere ligand.

**Scheme 5:**
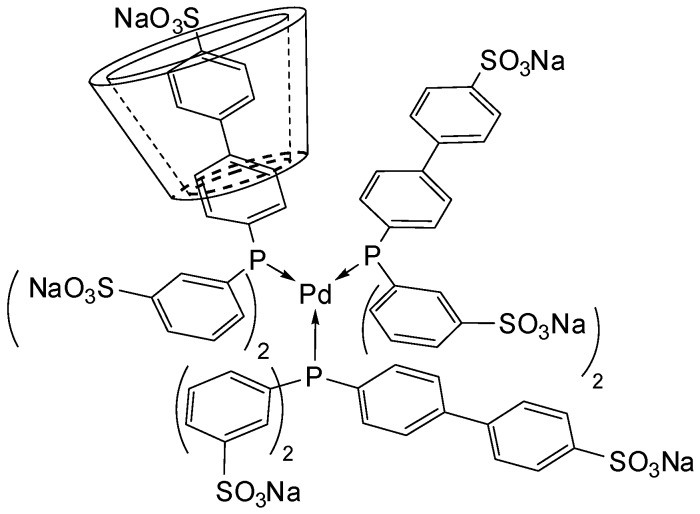
Possible geometry of the organometallic palladium species where CD plays the role of coordination second sphere ligand.

Furthermore, intense cross-peaks between the CD internal H_3_ protons and H_a_ of the biphenyl moiety together with high correlations between the CD internal H_5_ protons and H_c_/H_d_ of the biphenyl moiety in the T-ROESY spectrum allow us to propose a geometry where the phosphane penetration in the CD cavity takes place via the primary face (Scheme 5).

It should be noted however, that this geometry differs from the geometry of the inclusion complex between the free ligand in solution and the CD which takes place via the CD secondary face (see ESI). The spatial proximity of the palladium coordinated ligands inevitably leads to a high steric hindrance which could be responsible for such a difference in the geometry.

##### [Pd(0)/P(Biph)_2_PhTS] Complex

**Figure 12:**
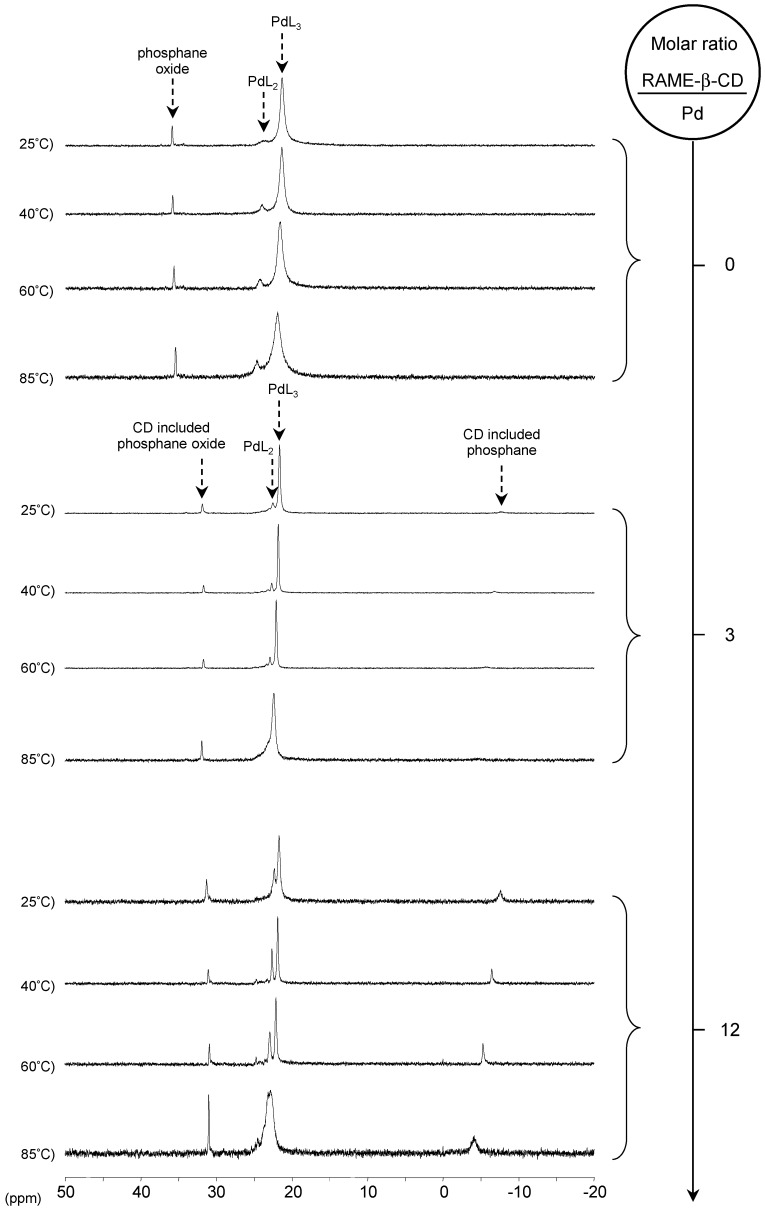
^31^P{^1^H} NMR spectra of palladium complexes synthetized with P(Biph)_2_PhTS depending on both temperature and RAME-β-CD quantity in D_2_O with a ligand concentration of 66 mM.

^31^P{^1^H} NMR spectra of Pd(P(Biph)_2_PhTS)_3_ complexes in addition to different RAME-β-CD concentrations and at different temperatures are given in Figure 12.

**Scheme 6:**
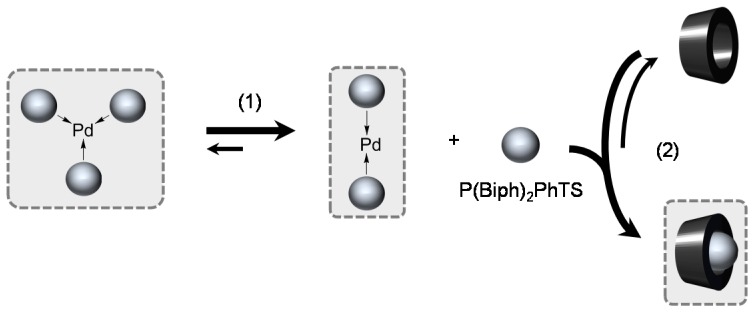
Equilibria between the different palladium species with P(Biph)_2_PhTS as ligand in the presence of 12 equiv. of RAME-β-CD toward palladium (species observed on the ^31^P{^1^H} NMR spectrum are presented on a grey background).

At room temperature and without CD, two different broad signals corresponding to the PdL_3_ and PdL_2_ complexes in fast equilibrium with free ligand are observed. Neither temperature, nor addition of 3 equiv. of RAME-β-CD do not allow to observe new organometallic species. Indeed, CD addition only leads to the apparition of the complexed free ligand at −7.5 ppm, a phosphane oxide chemical shift trapped by the CD and a sharpening of the organometallic complexes signals which are in equilibrium. However, addition of 12 equiv. of RAME-β-CD leads to a diminution of the PdL_3 _species in favour of the PdL_2_ species and free ligand trapped by the CD. On the other hand, temperature increase has a negligible influence on the PdL_3_/PdL_2_ ratio. At high temperature, natural dissociation increase of the PdL_3_ species is probably offset by the stability diminution of the inclusion complex between the CD and the phosphane. To sum up, high CD quantity is able to shift the equilibrium between the different palladium species towards the formation of low-coordinated species (Scheme 6).

##### [Pd(0)/P(Biph)_3_TS] Complex

**Figure 13:**
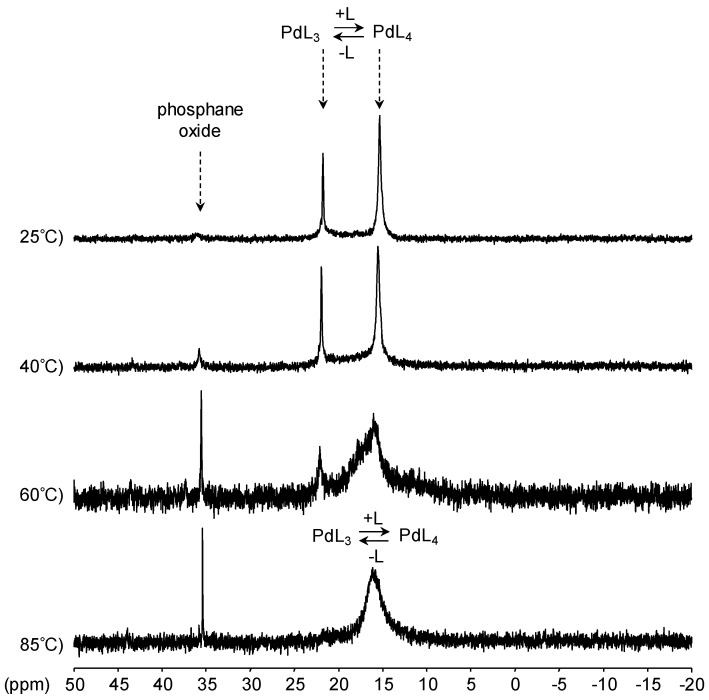
^31^P{^1^H} NMR spectra of palladium complexes synthetized with P(Biph)_3_TS depending on temperature in D_2_O with a ligand concentration of 66 mM.

As already described [16], the ligand P(Biph)_3_TS forms a new palladium species at room temperature:PdL_4_ which have a chemical shift of 15 ppm. Without CD, PdL_3_ and PdL_4_ species are in slow equilibrium. Indeed, when the temperature is increased, the signals of both complexes are broadened and merge together at 85 °C (Figure 13). Moreover, the NMR spectrum carried out at room temperature after heating gave the same signals reflecting the reversibility of the process.

**Figure 14:**
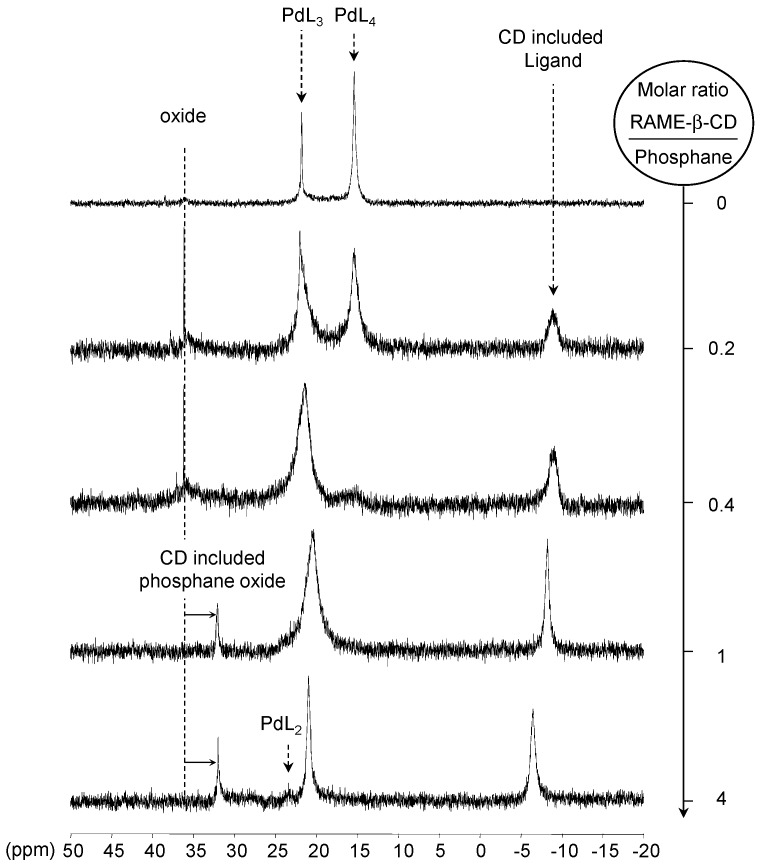
^31^P{^1^H} NMR spectra effect of RAME-β-CD quantity on the equilibrium of the different palladium species formed with the ligand P(Biph)_3_TS in D_2_O at 25 °C with a ligand concentration of 66 mM.

By adding increasing amounts of RAME-β-CD, the PdL_4_ signal disappears concurrently with an increase of both the PdL_3_ signal and the free ligand trapped by the CD (Figure 14).

**Figure 15:**
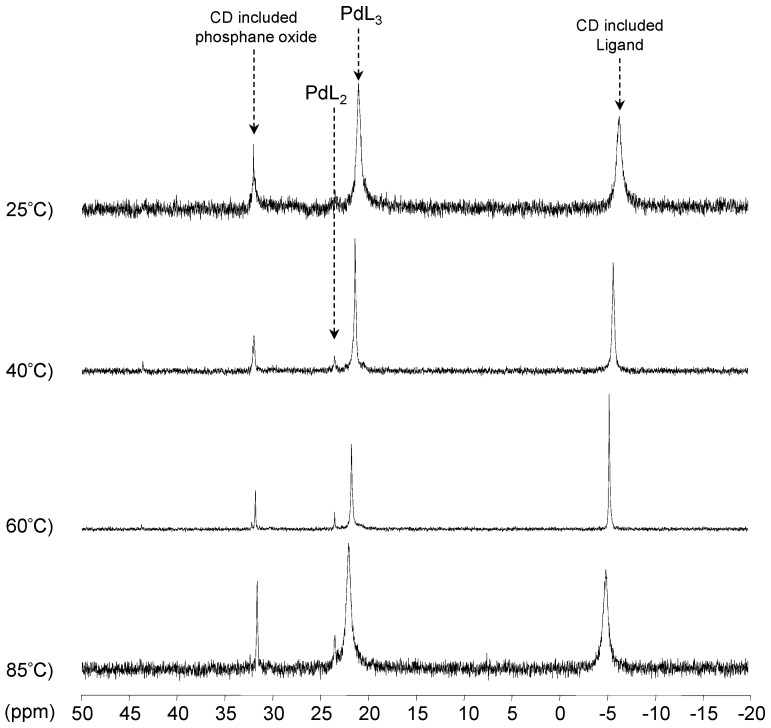
^31^P{^1^H} NMR spectra of palladium complexes synthetized with P(Biph)_3_TS depending on temperature in the presence of 4 equiv. of RAME-β-CD towards the phosphane in D_2_O with a ligand concentration of 66 mM.

When the CD quantity reaches four equivalents of ligand, species PdL_2_ appears. Similarly to the above-mentioned Pd(P(Biph)_2_PhTS)_3_ complex, temperature has a negligible influence on the PdL_3_/PdL_2_ ratio when P(Biph)_3_TS is used (Figure 15). Again, RAME-β-CD is able to shift the equilibria between the different palladium organometallic species when P(Biph)_3_TS is used as ligand.

A short overview of this palladium study is presented in Table 3. To sum up, the different ligands used in this study can be classified in three categories:

(1) ligands forming palladium complexes not affected by the RAME-β-CD addition (TPPTS, tris(*p*-Me)TPPTS and tris(*p*-OMe)TPPTS)
(2) ligands forming palladium complexes where the CD could play the role of coordination second sphere ligand (P(Biph)Ph_2_TS)
(3) ligands forming palladium complexes which are transformed by the presence of RAME-β-CD. Indeed, P(Biph)_2_PhTS and P(Biph)_3_TS spontaneously form respectively PdL_3_/PdL_2_ and PdL_4_/PdL_3_ organometallic complexes which are converted in PdL_2_ complexes when CD is added due to shift equilibria between the different organometallic species. However, formation of species where the CD acts as coordination second sphere ligand could not be avoided as the biphenyl moiety is well recognised by its cavity. In these cases, the organometallic complexes formed would naturally evolve to low-coordinated species through steric decompression by ligand decoordination.

### 2.3. Comparison of the Results Obtained with Palladium and Platinum Complexes

An overview of the results is presented in Table 4.

Firstly, tris*-ortho-*substituted hydrosoluble phosphanes led to low coordinated platinum species and are unable to extract palladium from a Pd(TPP)_4_ toluene solution (Table 4, entry 3). These two behaviours are probably due to the steric hindrance around their phosphorous atom making them poor ligands. Apart from this peculiar case and the results obtained with platinum for P(Biph)_2_PhTS and P(Biph)_3_TS ligands (broad signals in ^31^P{^1^H} NMR) (entries 5 and 6), three CD behaviours towards [PtL_3_Cl]Cl and PdL_n_ complexes were noticed:

- The CD can have no influence on the organometallic species. That is what is observed with *para*-substituted hydrosoluble phosphanes for both palladium and platinum complexes (entry 2) and also with TPPTS, only in the case of palladium (entry 1). For the *para*-substituted hydrosoluble phosphanes, this behaviour could be explained by the lack of interaction between the CD and the phosphane (tris(*p*-OMe)TPPTS), or by a too strong metal-phosphorus bond coming from a high σ-donor ligand (tris(*p*-Me)TPPTS). However, the fact that the PdL_2_ species is not formed after CD addition on the PdL_3_ complex not only for tris(*p*-OMe)TPPTS and tris(*p*-Me)TPPTS but also for TPPTS probably comes from the too unstable PdL_2_ (14e^−^ species) in the case of TPPTS derivatives.
- The CD can act as a phosphane decoordination promotor. This property was emphasised with TPPTS as ligand in the [PtL_3_Cl]Cl complex (entry 1), and with P(Biph)_2_PhTS and P(Biph)_3_TS ligands in the case of PdL*_n_* type complexes (entries 5 and 6). The fact that TPPTS can be removed from Pt by addition of CD, contrary to tris(*p*-OMe)TPPTS and tris(*p*-Me)TPPTS, comes not only from its ability to be included in the CD cavity but also to its lower coordination power (less basic ligand).
- The CD can act as a second sphere ligand by including the metal-bound phosphane. This property was undoubtedly highlighted for both platinum and palladium complexes with the P(Biph)Ph_2_TS ligand whose *p*-sodiosulfobiphenyl group is well recognized by the CD. Because of this identical group present in their structure, making them accessible for a cyclodextrin even when bound to a metal, it is also important to say that P(Biph)_2_PhTS and P(Biph)_3_TS based organometallic species form also surely supramolecular complexes with CD. This property plays probably a role in the observed decoordination of P(Biph)_2_PhTS and P(Biph)_3_TS from palladium, the CD being able to stabilize the low-coordinated resulting organometallic species. More concretely, this property can explain the observation of PdL_2_ type species when P(Biph)_3_TS ligand was used, species not formed with TPPTS ligand. Indeed, TPPTS structure doesn’t permit complexation when bound to the metal.

The three evoked behaviours of CD in the presence of a phosphane bound to a metal depend on different equilibria whose evolution is assumed to be under thermodynamic control. Indeed, in all the experiments conducted in this study, the temperature had always a reversible effect on the system. More concretely, the modification of spectra sometimes observed by heating disappeared systematically by recovering the room temperature.

**Scheme 7:**
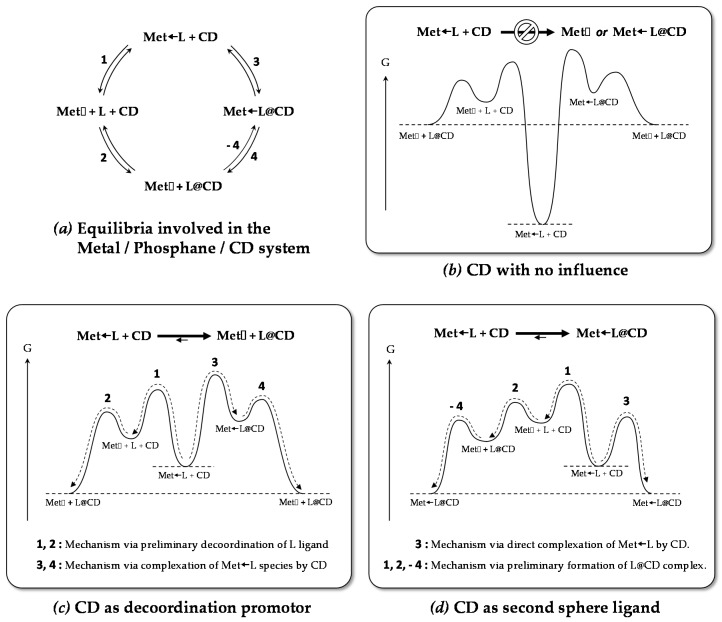
Different possible mechanisms for the cyclodextrin assisted generation of new organometallic species from a metal coordinated by a ligand (Met ← L species); Met = metal, L = Phosphane, L@CD = ligand included in the CD cavity.

Basically, by considering a simplified system consisting of a cyclodextrin (CD) and a phosphane (L ligand) coordinated to a metal (Met), i.e., a “Met ← L + CD” system, several evolutions are possible. Firstly, the phosphane can naturally leave the metal by decoordination and lead to a “Met**⌷** + L + CD” system (Scheme 7a, 1). This last system can then be transformed by inclusion of the free ligand in the CD cavity (Scheme 7a, 2). Starting from the same initial system (“Met ← L + CD”), another evolution can be the complexation of ligand bound to the metal to reach a “Met ← L@CD” system in which the CD acts as a second sphere ligand (Scheme 7a, 3). In this complex, the “L@CD” part constitutes a supramolecular ligand which can therefore also leave the metal to lead to a “Met**⌷** + L@CD” system (Scheme 7a, 4). The fore-mentioned systems are in equilibrium and their proportions are consequently driven by their free enthalpy, the more stable system being the more represented one.

So, when the “Met ← L” starting organometallic species stays perfectly stable in the presence of CD, it means that the free enthalpy of the “Met ← L + CD” system is very low compared to the other possible systems (Scheme 7b). Different reasons can produce this situation: basicity of the ligand making the “Met ← L” bond very strong, ligand poorly recognized by the CD...

In the same way, when CD is proved to be a decoordination promotor by being able to do an inclusion complex with the phosphane, it means that the “Met**⌷** + L@CD” system is relatively close to the “Met ← L + CD” initial system, in terms of free enthalpy (Scheme 7(c)). In this case, the “Met**⌷** + L@CD” system can be reached either by preliminary natural decoordination of the phosphane via the “Met**⌷** + L + CD” system (Scheme 7(c)), mechanism with the successive steps 1 and 2) or by preliminary complexation of the organometallic species by the CD (Scheme 7(c), mechanism with steps 3 and 4 successively). This second mechanism is probably favoured when the CD is able to include the ligand bound to the metal, i.e., when the free enthalpy of the Met ← L@CD is low. Therefore, one can think that TPPTS decoordination from [PtL_3_Cl]Cl species by CD proceed via the “1, 2 “ mechanism (Met ← L@CD species is too high in free enthalpy to be easily reached in the case of TPPTS) and that biphenyl phosphanes decoordination from palladium species proceed by the “3, 4” mechanism (Met ← L@CD is easy to form).

Finally, the “Met ← L@CD” species can be obtained from the initial “Met ← L + CD” system via direct complexation (scheme 7(d), step 3) or via preliminary formation of the “L@CD” inclusion complex in solution (Scheme 7(d), steps 1, 2 and -4).

**Scheme 8:**
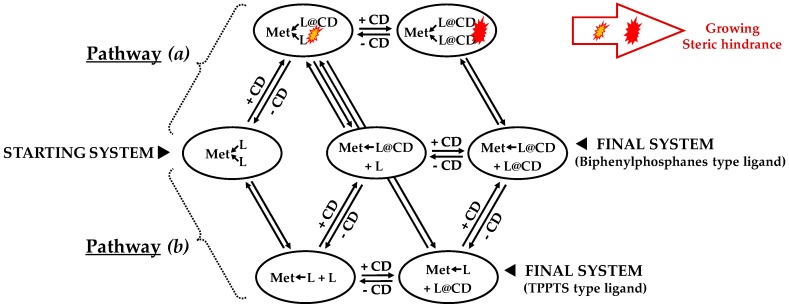
Two possible pathways of generation of low coordinated organometallic species thanks to a cyclodextrin from a species bearing several ligands (symbolized by “MetL_2_”): (**a**) via supramolecular organometallic species; (**b**) via preliminary decoordination of a ligand; Met = metal, L = Phosphane, L@CD = ligand included in the CD cavity.

The simple rationalization of the different transformations observed in this study for platinum and palladium complexes in the presence of RAME-β-CD can be refined by taking into account the fact that several ligands are systematically present on the metal. In particular, for ligands allowing a complexation by CD when coordinated to the metal (biphenyl phosphanes, in this study), relatively stable organometallic species possessing two spheres of coordination (phosphane and CD, respectively) may be obtained. However, in these species, the steric hindrance generated by the complexation by the CD constitutes a possible reason explaining that the included ligand or a neighbour ligand can be expulsed to reach a more stable system (Scheme 8, pathway (a)).

Conversely, for ligands not accessible for a CD when bound to a metal (TPPTS, in this study), the only way to explain an observed decoordination in the presence of CD is undoubtedly the preliminary decoordination of the ligand from the metal (Scheme 8, pathway (b)).

## 3. Materials and Methods

### 3.1. General Remarks

The ^1^H and ^31^P{^1^H} NMR spectra were recorded on an Avance 300 DPX instrument (Bruker, Karlsruhe, Germany) at 300.13 and 121.49 MHz, respectively. Organic compounds were purchased from Fisher Scientific (Pittsburgh, PA, USA) in their highest purity and used without further purification. Potassium tetrachloroplatinate(II) and tetrakis(triphenylphosphane)palladium(0) were purchased from Strem Chemicals (Newburyport, MA, USA). Ultrapure water was used in all experiments (Fresenius Kabi, Bad Homburg, Germany; γ = 72.0 mN.m^−1^ at 25 °C). Pharmaceutical grade RAME-β-CD (Cavasol^®^ W7 M) was purchased from Wacker Chemie GmbH (München, Germany) and was used as received. Randomly methylated β-cyclodextrin (RAME-β-CD) is a partially methylated cyclodextrin and its degree of substitution was equal to 1.8 (1.8 methyl groups per glucopyranose unit). TPPTS [24], tris(*o*-Me)TPPTS [25], tris(*o*-OMe)TPPTS [25], tris(*p*-Me)TPPTS [15] and tris(*p*-OMe)TPPTS [26], P(Biph)Ph_2_TS [16], P(Biph)_2_PhTS [16] and P(Biph)_3_TS [16] were prepared as reported in the literature. The purity of these water-soluble phosphanes was controlled by ^1^H, ^13^C{^1^H} and ^31^P{^1^H} NMR analysis.

### 3.2. ^31^P{^1^H} NMR Study on Platinum and Palladium Complexes

Platinum complexes were synthetized by dissolving K_2_PtCl_4_ (8.3 mg, 0.02 mmol) in degassed deuterated water (2 mL). Three equiv. of the corresponding ligand (0.06 mmol) were added to the solution, which was then stirred for 15 min at room temperature under nitrogen. Studies in the presence of RAME-β-CD were conducted as follows: the required amount of cyclodextrin was introduced into 500 μL of the above solution. After 15 min of stirring, the solution was transferred into a NMR tube and analysed.

Palladium complexes were synthetized according to a modified literature procedure [27]. Pd(PPh_3_)_4_ (103 mg, 0.089 mmol.) was dissolved in 2 g of degassed toluene into a Schlenk tube under nitrogen. The corresponding phosphane (1.5 equiv., 0.133 mmol) was dissolved in 2 g of D_2_O and cannulated onto the palladium solution. The mixture was stirred for 30 min at room temperature. After decantation, the organic phase was removed and a new degassed Pd(PPh_3_)_4_ solution was added to the previous aqueous solution which underwent again a 30 min stirring period to ensure an optimal extraction of the palladium by the hydrosoluble ligand. After decantation, the aqueous phase was recovered. The obtained PdL_n_ (*n* = 2, 3 or 4) solution was then used for the ^31^P{^1^H} NMR study. The previous solution usually contained a small excess of free ligand. Study in the presence of RAME-β-CD was conducted as follow: to 1 mL of the above solution was introduced under nitrogen the required amount of RAME-β-CD. After 15 min of stirring, the solution was transferred via cannula into a nitrogen pressurized 5 mm NMR tube.

## 4. Conclusions

A ^31^P{^1^H} NMR study of platinum(II) and palladium(0) complexes coordinated with two different groups of hydrosoluble monodentate phosphane ligands (including TPPTS, *meta*-trisulfonated triphenylphosphane derivatives bearing one methyl (or methoxy) group on the aromatic ring and trisulfonated biphenylphosphanes) has been described. The effect of randomly methylated β-cyclodextrin (RAME-β-CD) addition on these complexes has been examined.

It has been shown that the interaction of the free phosphane with RAME-β-CD is a prerequisite to observe any effect of the CD on the organometallic species (case of tris(*p*-OMe)TPPTS: no interaction, thus no effect). However, the stereoelectronic properties of the ligand are also of great importance. On the one hand, ligands whose phosphorous atom is hindered by *ortho*-substituted *meta*-sulfophenyl groups lead, without CD, to low coordinated organometallic species, even with an excess of ligand; the lack of free volume in the coordination sphere of these species doesn’t permit the coordination of another ligand (case of tris(*o*-Me)TPPTS and tris(*o*-OMe)TPPTS, with platinum). On the other hand, a high σ-donating ligand can be difficult to be removed from the metal by CD because of the stability of its metal-phosphorous bond (case of tris(*p*-Me)TPPTS, with platinum as well as palladium).

More generally, two interesting behaviours of CD have been highlighted: the CD can act as a second sphere ligand by including the metal-bound phosphane without denaturing the organometallic species or as a phosphane decoordination promotor. These two behaviours are under thermodynamic control. Depending on the used ligand structure, the decoordination observed in the presence of CD may proceed either via a preliminary decoordination of the phosphane followed by a complexation of the free ligand by the CD (case of TPPTS) or via the generation of organometallic species complexed by CD which then lead to expulsion of ligands to decrease their internal steric hindrance (cases of biphenylphosphanes).

Such modifications of organometallic complexes by a CD, i.e., inclusion of organometallic species into the cyclodextrin cavity by one of its ligand or decoordination of a ligand from the metal, can be efficient strategies to obtain new catalysts possessing original activities and selectivities.

## Figures and Tables

**Table 1:** Structure, abbreviation of the water-soluble phosphanes used, and value of the association constant (K) for the inclusion complexes between the RAME-β-CD and the phosphane.

Entry	Structure	Name Abbreviation	K (M^−1^) ^a,b^
1	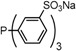	tris(3-sodium-sulfonatophenyl)phosphane TPPTS	840 [15]
2	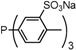	tris(4-methyl-3-sodium-sulfonatophenyl)phosphane tris(*p*-Me)TPPTS	960 [15]
3	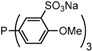	tris(4-methoxy-3-sodium-sulfonatophenyl)phosphane tris(*p*-OMe)TPPTS	0 [15]
4	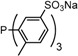	tris(6-methyl-3-sodium-sulfonatophenyl)phosphane tris(*o*-Me)TPPTS	0 [15]
5	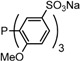	tris(6-methoxy-3-sodium-sulfonatophenyl)phosphane tris(*o*-OMe)TPPTS	0 [15]
6	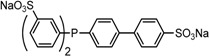	trisulfonated monobiphenyldiphenylphosphane P(Biph)Ph_2_TS	9900 ^c^
7	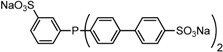	trisulfonated bisbiphenylmonophenylphosphane P(Biph)_2_PhTS	>100,000 ^c^
8	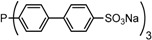	trisulfonated trisbiphenylphosphane P(Biph)_3_TS	>100,000 ^c^

^a^ In each case, the stoichiometry has been determined to be equal to 1:1 when an inclusion complex was formed; ^b^ K constant is an average constant due to the random methylation of the CD; ^c^ association constant determined between native β-CD and the phosphane.

**Table 2:** Organometallic species formed by adding 3 equiv. of ligand to a K_2_PtCl_4_ aqueous solution (10 mM) and after RAME-β-CD addition (1 to 12 equiv. toward platinum).

Ligand	Platinum Complex Obtained without CD	New Species Obtained after RAME-β-CD Addition
TPPTS	[PtL_3_Cl]Cl = 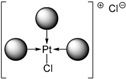	*cis*-PtCl_2_L_2_ = 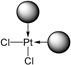
tris(*p*-Me)TPPTS	[PtL_3_Cl]Cl	-
tris(*p*-OMe)TPPTS	[PtL_3_Cl]Cl	-
tris(*o*-Me)TPPTS	*trans*-PtCl_2_L_2_ = 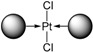	-
tris(*o*-OMe)TPPTS	*trans*-PtCl_2_L_2_	-
P(Biph)Ph_2_TS	[PtL_3_Cl]Cl	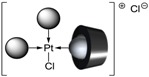 or 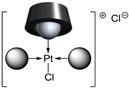
P(Biph)_2_PhTS	n.d (broad signals) *	n.d (broad signals)
P(Biph)_3_TS	n.d (broad signals) *	n.d (broad signals)

* Probably [PtL_3_Cl]Cl when compared with spectrum obtained with P(Biph)Ph_2_TS.

**Table 3:** New organometallic species observed after RAME-β-CD addition (1 to 12 equiv. toward palladium) to a solution of the palladium complexes formed by extraction with an aqueous ligand solution (66 mM).

Ligand	Palladium Complex Obtained without CD	New Species Obtained after RAME-β-CD Addition
TPPTS	PdL_3_	-
tris(*p*-Me)TPPTS	PdL_3_	-
tris(*p*-OMe)TPPTS	PdL_3_	-
tris(*o*-Me)TPPTS	No palladium extraction	-
tris(*o*-OMe)TPPTS	No palladium extraction	-
P(Biph)Ph_2_TS	PdL_3_	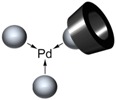
P(Biph)_2_PhTS	PdL_3_ + PdL_2_	[PdL_2_] increase
P(Biph)_3_TS	PdL_4_ + PdL_3_	[PdL_3_] increase + PdL_2_ formed

**Table 4:** Effects of RAME-β-CD addition on the platinum and palladium complexes obtained with the different phosphanes (in D_2_O, at 25 °C); CD = RAME-β-CD; L@CD = Ligand included in the CD cavity.

Entry	Ligand (L)	Influence of CD on Pt Complexes	Influence of CD on Pd Complexes
1	TPPTS	[PtL_3_Cl]Cl + CD → *cis*-PtCl_2_L_2_ + L@CD CD = decoordination promotor	PdL_3_ unchanged with CD
2	tris(*p*-Me)TPPTS tris(*p*-OMe)TPPTS	[PtL_3_Cl]Cl unchanged with CD	PdL_3_ unchanged with CD
3	tris(*o*-Me)TPPTS tris(*o*-OMe)TPPTS	[PtL_3_Cl]Cl impossible to obtain; *trans*-PtCl_2_L_2_ was formed with or without CD	Ligand unable to extract Pd from the organic layer with or without CD
4	P(Biph)Ph_2_TS	[PtL_3_Cl]Cl + CD → [(L@CD)PtL_2_Cl]Cl CD = second sphere ligand	PdL_3_ + CD → (L@CD)PdL_2_ CD = second sphere ligand
5	P(Biph)_2_PhTS	n.d. (broad signals with or without CD)	PdL_3_ + CD → PdL*_2_ + L@CD L* = L or L@CD CD = decoordination promotor and second sphere ligand
6	P(Biph)_3_TS	n.d. (broad signals with or without CD)	PdL_4_ + CD → PdL*_3_ + L@CD PdL*_3_ + CD → PdL*_2_ + L@CD L* = L or L@CD CD = decoordination promotor and second sphere ligand

## Supplementary Materials

The following are available online at http://www.mdpi.com/1420-3049/22/1/140/s1: Figures S1–S12.
