# Supplementary material for: Transition Metal Complexes Coordinated by Water Soluble Phosphane Ligands: How Cyclodextrins Can Alter the Coordination Sphere?
Source: Molecules. 2017 Jan 17;22(1):140. doi: 10.3390/molecules22010140 (PMC6155594; doi:10.3390/molecules22010140)
Supplement: Supplementary file 1 [file molecules-22-00140-s001.pdf]

Supplementary Materials: Transition Metal Complexes Coordinated by Water Soluble Phosphane Ligands: How Cyclodextrins can Alter the Coordination Sphere?

Michel Ferreira, Hervé Bricout, Sébastien Tilloy and Eric Monflier

1. Platinum Conversion Determination

When RAME-β-CD is added to an aqueous solution of the pure $[PtL_3Cl]^+$, Cl^- organometallic complex, species $PtCl_2L_2$ could be formed according to (L = water-soluble phosphane):

$$[PtL_3Cl]^+, Cl^- \rightarrow PtCl_2L_2 + L \quad (1)$$

Platinum conversion could be written as follows:

$$conv_{Pt}\,(\%) = \frac{n_{PtCl_2L_2}}{n_{[PtL_3Cl]^+,Cl^-} + n_{PtCl_2L_2}} \times 100 \quad (2)$$

where n_x = x mole number after RAME-β-CD addition.

Taking into account the phosphorus atoms number in the platinum species, Equation (2) becomes:

$$conv_{Pt}\,(\%) = \frac{\frac{S_{PtCl_2L_2}}{2}}{\frac{S_{[PtL_3Cl]^+,Cl^-}}{3} + \frac{S_{PtCl_2L_2}}{2}} \times 100 \quad (3)$$

where S_x = $^{31}P\{^1H\}$ NMR total surface relating to species x.

The fact that $PtCl_2L_2$ species often lead to a large signal difficult to integrate, combined with satellites signals (coming from the platinum coupling) close to the background noise, led us to use the M_d (doublet) and M_L (singlet) signals of the $^{31}P\{^1H\}$ NMR spectrum represented Figure S1 to calculate the platinum conversion (in order to clarify, the $PtCl_2L_2$ large signals were not represented). The M_L singlet corresponds to the decoordinated phosphane ligand coming from the cyclodextrin addition. S_d and S_L are respectively the surface area of the M_d and M_L signals.

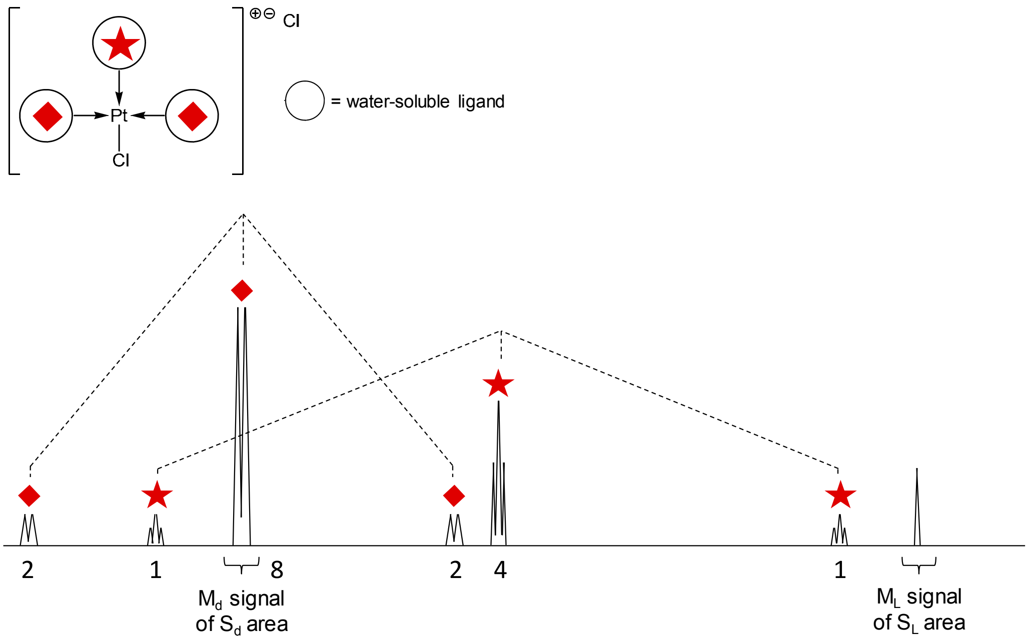

Figure S1. Theoretical $^{31}P\{^1H\}$ spectrum of $[PtL_3Cl]^+$, Cl^- and free ligand signal (L).

As only one third of the platinum (^{195}Pt) has a spin of 1/2, doublets and triplets of the $[PtL_3Cl]^+$, Cl^- species appear, from downfield to upfield, in relative ratios 2:1:8:2:4:1, representing a total area of 18. So:

$$S_d = \frac{8}{18} \times S_{[PtL_3Cl]^+,Cl^-} = \frac{4}{9} \times S_{[PtL_3Cl]^+,Cl^-} \quad (1)$$

Moreover, given that every ligand decoordination from the $[PtL_3Cl]^+$, Cl^- complex leads to formation of 1 equiv of $PtCl_2L_2$:

$$S_L = \frac{S_{Cl_2PtL_2}}{2} \quad (2)$$

By combining Equations (4) and (5) with (3):

$$\mathrm{conv}_{Pt}\,(\%) = \frac{S_L}{\frac{3\,S_d}{4} + S_L} \times 100 \quad (3)$$

2. Complexes formed by Mixing K_2PtCl_4 Platinum Aqueous Solution with 2 Equiv. of *Para*-Substituted Phosphane

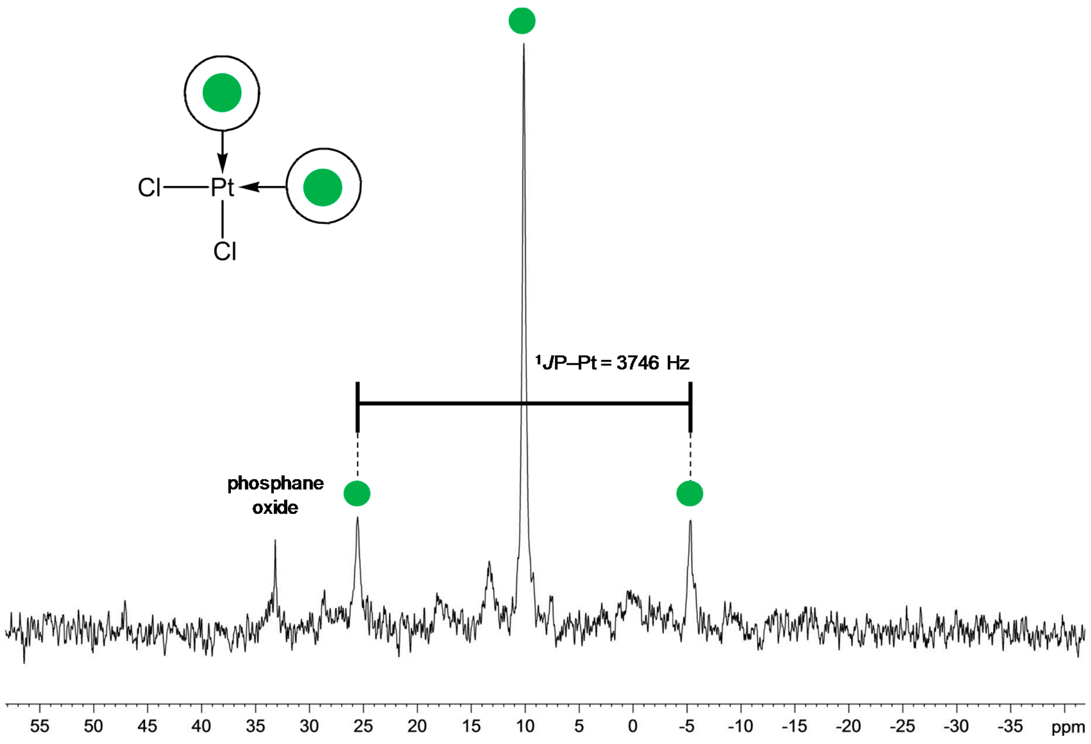

Figure S2. ^{31}P{^{1}H}-NMR spectrum of *cis*-$PtCl_2$(tris(*p*-Me)TPPTS)$_2$ at 25 °C.

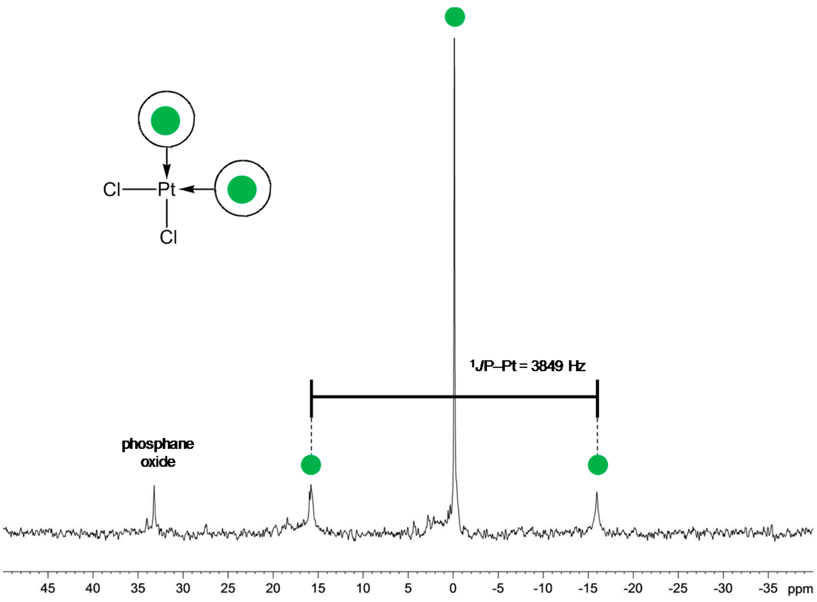

Figure S3. $^{31}P\{^1H\}$-NMR spectrum of *cis*-$PtCl_2$(tris(*p*-OMe)TPPTS)$_2$ at 25 °C.

3. RAME-β-CD Influence on $[PtL_3Cl]Cl$ Complexes with L = tris(*p*-Me)TPPTS and L = tris(*p*-OMe)TPPTS

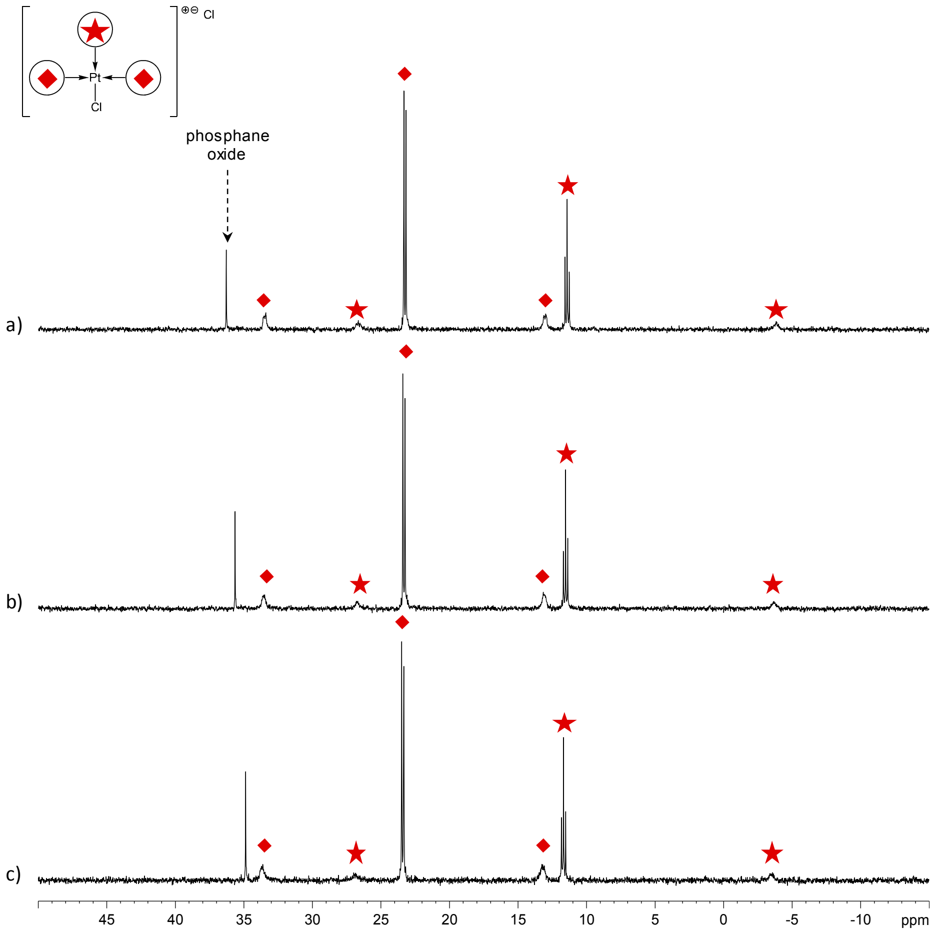

Figure S4. $^{31}P\{^1H\}$-NMR spectrum of (**a**) [Pt(tris(*p*-Me)TPPTS)$_3$Cl]Cl at 25 °C, with addition of (**b**) 3 equiv. or (**c**) 9 equiv. of RAME-β-CD.

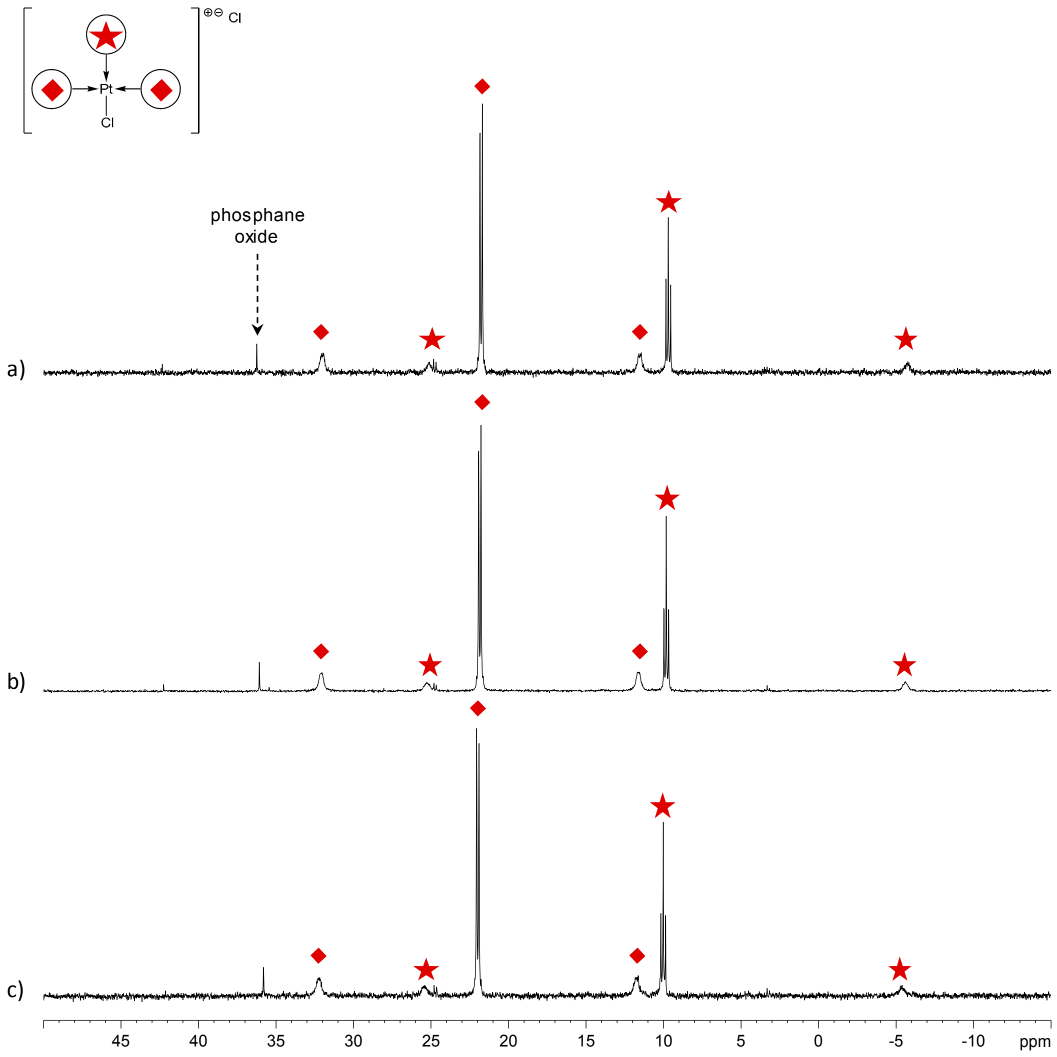

Figure S5. $^{31}P\{^{1}H\}$-NMR spectrum of (**a**) [Pt(tris(*p*-OMe)TPPTS)$_3$Cl]Cl at 25 °C, with addition of (**b**) 3 equiv. or (**c**) 9equiv. of RAME-β-CD.

4. Interaction Study β-CD/Biphenylphosphanes

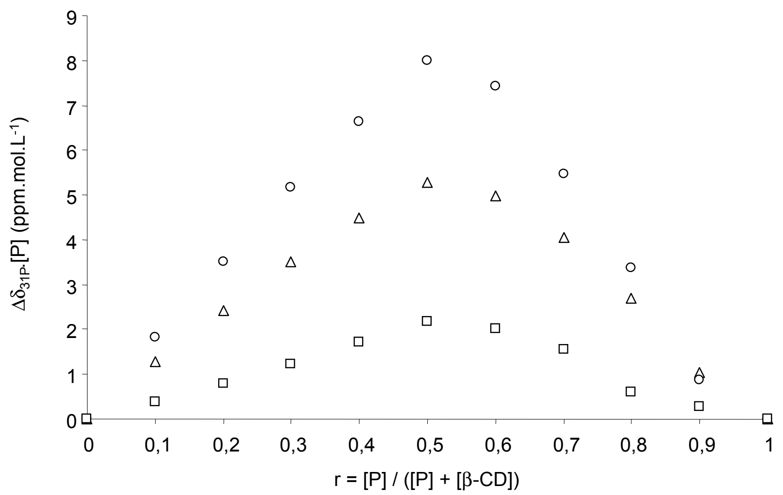

Figure S6. Job plots for inclusion complexes β-CD/biphenyl phosphane at 25 °C in D_2O. P(Biph)$_2$PhTS: ○; de la P(Biph)Ph$_2$TS: Δ; de la P(Biph)$_3$TS: □.

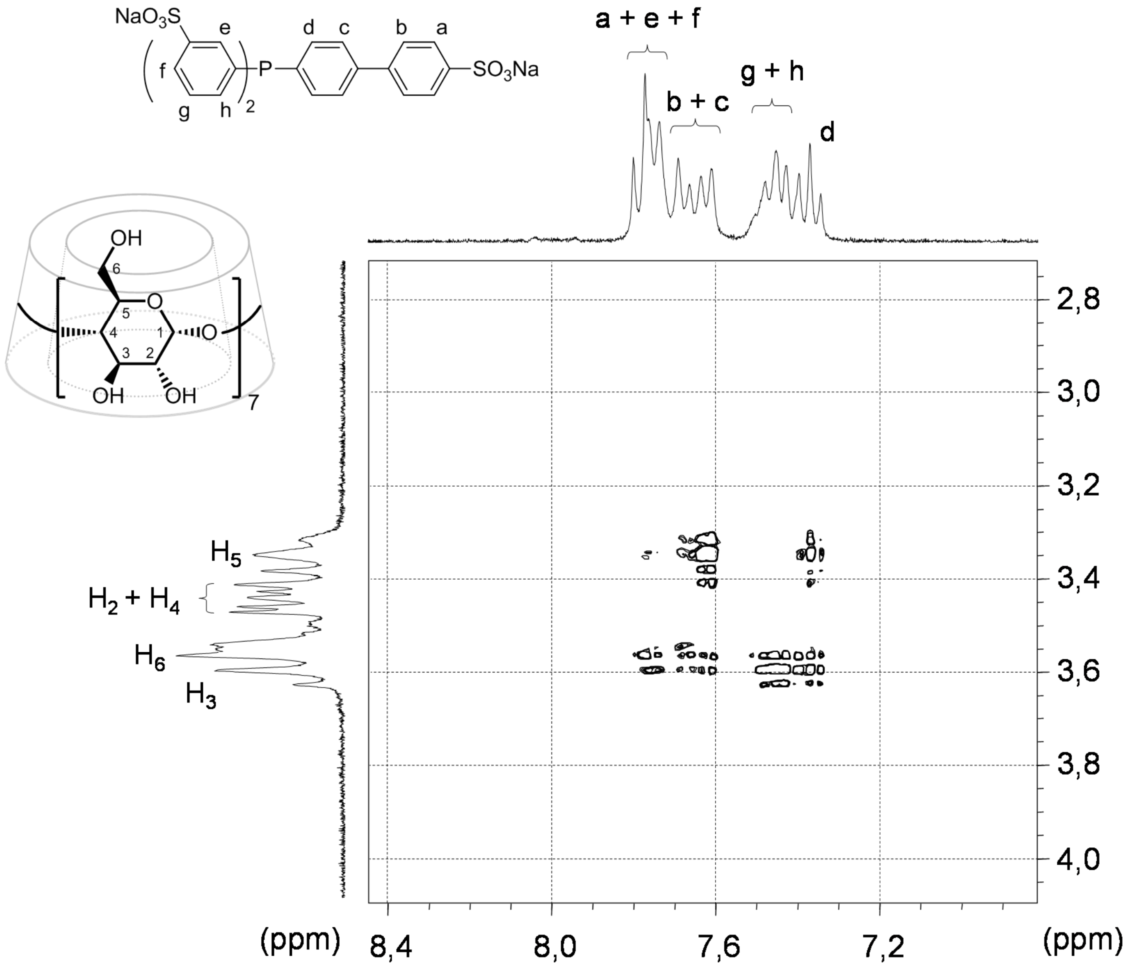

Figure S7. 2D T-ROESY spectrum of a solution containing [β-CD] (1 mM) and [P(Biph)Ph2TS] (4 mM) at 25 °C in D2O.

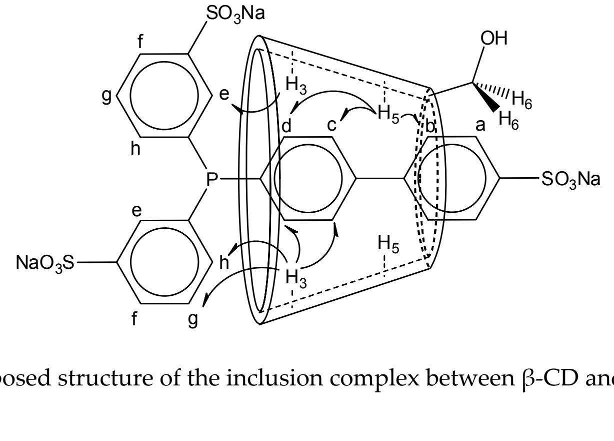

Figure S8. Proposed structure of the inclusion complex between β-CD and P(Biph)Ph2TS.

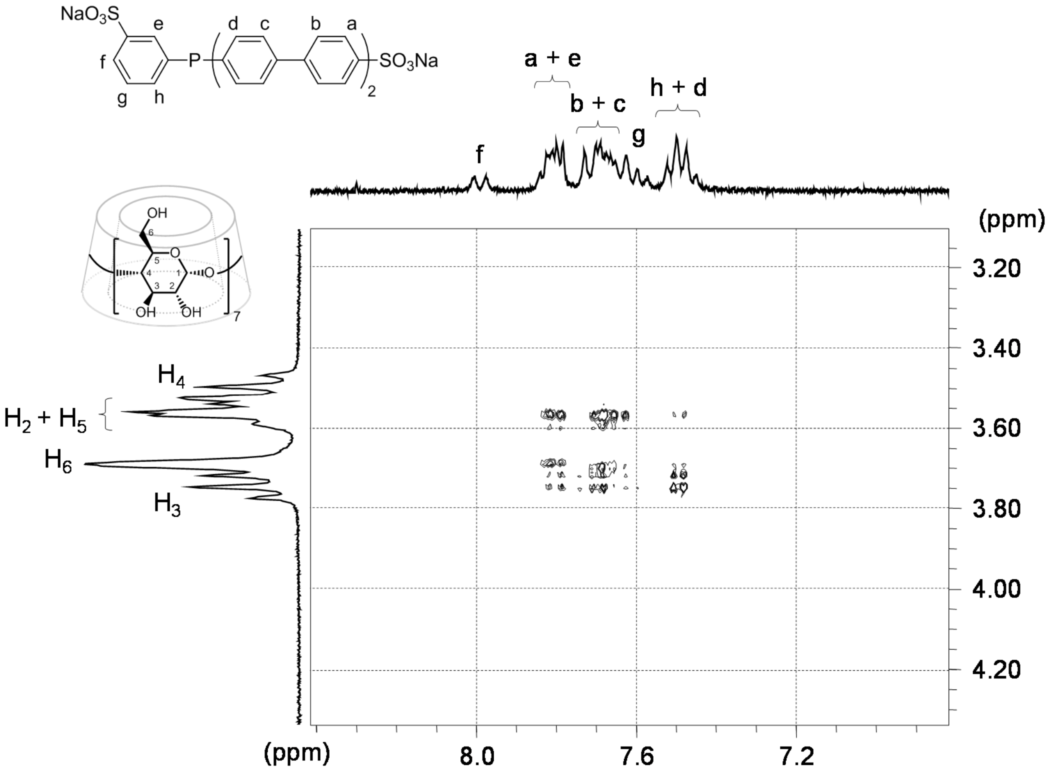

Figure S9. 2D T-ROESY spectrum of a solution containing [β-CD] (7 mM) and [P(Biph)$_2$PhTS] (3 mM) at 25 °C in D_2O.

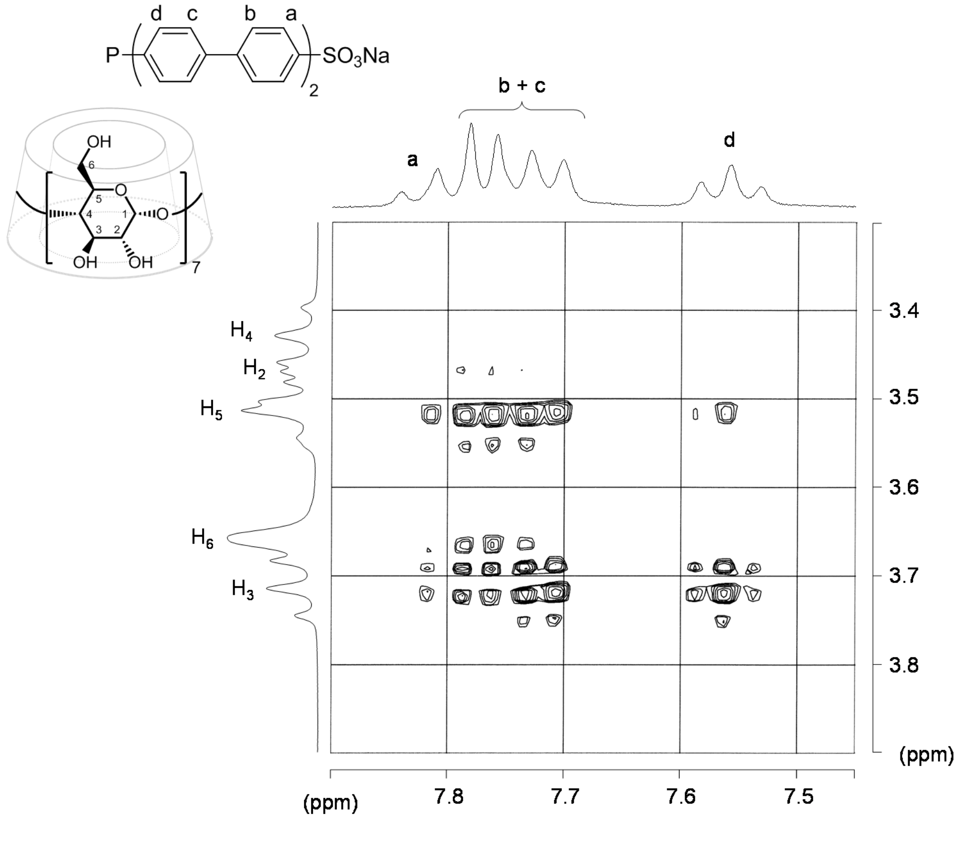

Figure S10. 2D T-ROESY spectrum of a solution containing [β-CD] (7 mM) and [P(Biph)$_3$TS] (3 mM) at 25 °C in D_2O.

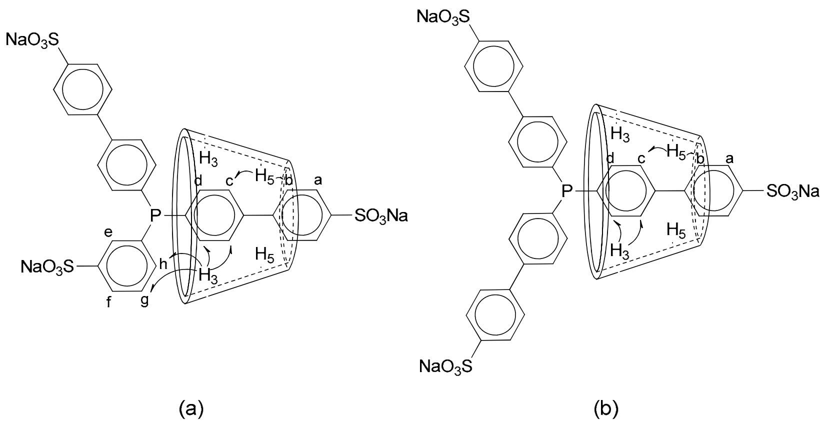

Figure S11. Proposed structure of the inclusion complex between β-CD and (**a**) P(Biph)$_2$PhTS. or (**b**) P(Biph)$_3$TS.

5. RAME-β-CD Influence on PdL$_3$ Complex with L = P(Biph)Ph$_2$TS

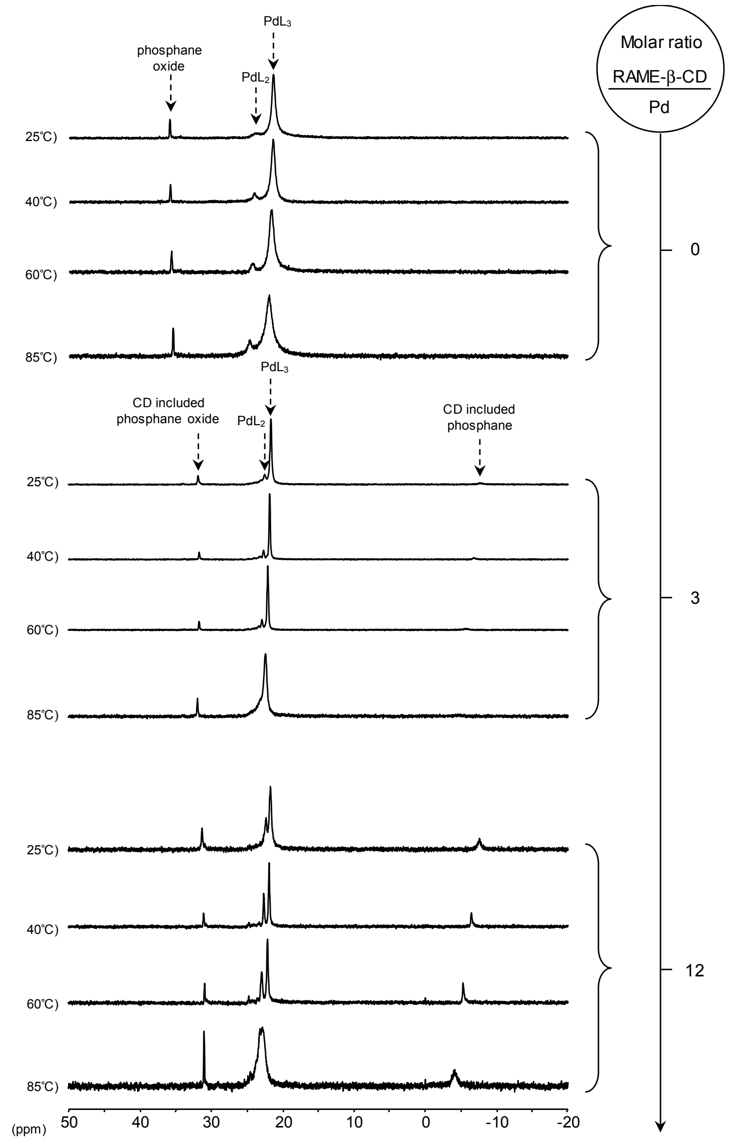

Figure S12. $^{31}P\{^{1}H\}$-NMR spectra of Pd(P(Biph)Ph$_2$TS)$_3$ depending on both temperature and RAME-β-CD quantity in D_2O with a ligand concentration of 66 mM.
